# Candidate Gene Association Studies of Anthracycline-induced Cardiotoxicity: A Systematic Review and Meta-analysis

**DOI:** 10.1038/s41598-017-00075-1

**Published:** 2017-02-27

**Authors:** Siew Lian Leong, Nathorn Chaiyakunapruk, Shaun Wen Huey Lee

**Affiliations:** 1 School of Pharmacy, Monash University Malaysia, Jalan Lagoon Selatan, Bandar Sunway, 46150 Selangor Malaysia; 2 Faculty of Pharmacy, Cyberjaya University College of Medical Sciences, Cyberjaya, 63000 Selangor Malaysia; 3 Center of Pharmaceutical Outcomes Research (CPOR), Department of Pharmacy Practice, Faculty of Pharmaceutical Sciences, Naresuan University, Phitsanulok, Thailand; 4 School of Pharmacy, University of Wisconsin, Madison, USA; 5 School of Population Health, University of Queensland, Brisbane, Australia

## Abstract

Anthracyclines play an important role in the management of patients with cancer but the development of anthracycline-induced cardiotoxicity (ACT) remains a significant concern for most clinicians. Recently, genetic approach has been used to identify patients at increased risk of ACT. This systematic review assessed the association between genomic markers and ACT. A systematic literature search was performed in Medline, PubMed, Cochrane Central Register of Controlled Studies, CINAHL Plus, AMED, EMBASE and HuGE Navigator from inception until May 2016. Twenty-eight studies examining the association of genetic variants and ACT were identified. These studies examined 84 different genes and 147 single nucleotide polymorphisms. Meta-analyses showed 3 risk variants significantly increased the risk for ACT; namely ABCC2 rs8187710 (pooled odds ratio: 2.20; 95% CI: 1.36–3.54), CYBA rs4673 (1.55; 1.05–2.30) and RAC2 rs13058338 (1.79; 1.27–2.52). The current evidence remains unclear on the potential role of pharmacogenomic screening prior to anthracycline therapy. Further research is needed to improve the diagnostic and prognostic role in predicting ACT.

## Introduction

Anthracycline antibiotics are among the most potent chemotherapeutic agents since their introduction 50 years ago. Agents in this pharmacological group of antineoplastic drugs include doxorubicin, daunorubicin, epirubicin, and idarubicin. They are the backbone for many chemotherapy regimens in the treatment of breast cancer^1, 2^, lymphoma^3–7^, leukaemia^8, 9^ and sarcomas^10, 11^. This may be due to the wide range of mechanisms which anthracyclines are thought to act on including: (i) initiation of apoptosis via inhibition of topoisomerase II, (ii) DNA synthesis inhibition, (iii) DNA binding and alkylation, (iv) DNA cross-linking, (v) interference with DNA strand separation and helicase activity, and (vi) free radical formation and lipid peroxidation^12^. While anthracyclines have revolutionised the management of both early and advance-stage diseases, the clinical usefulness of anthracyclines is compromised by the adverse effects of cardiac toxicity. Regimens using anthracyclines were reported to increase the risk of clinical and subclinical cardiac toxicity as well as death by more than 5-fold^13–15^.

Thus, the early identification of patients at risk of cardiotoxicity is a primary goal for many cardiologist and oncologist. Research over the past few decades have identified several risk factors associated with ACT including: aged ≥ 65 years old or less than 4 years old, female gender, pre-existing hypertension and/or cardiac disease, mediastinal radiation, high doses of anthracycline as well as concurrent treatment with cyclophosphamide, paclitaxel and trastuzumab^16, 17^. Nevertheless, most of these approaches have low diagnostic sensitivity and predictive power to detect subclinical myocardial injury^18, 19^. Several studies have recently reported the use of genetic variants as prognostic biomarkers for early detection of ACT^20–23^. The aim of the current study was to provide an overview on studies using genetic markers for identification of patients at risk of ACT and summarise these associations.

## Methods

### Search strategy

We searched OVID Medline, PubMed, Cochrane Central Register of Controlled Studies, CINAHL Plus, AMED, EMBASE and HuGE Navigator from inception until May 2016. The search terms include anthracycline, cardiotoxicity and genetic (The full search term can be found in Supplementary Information: Search Strategies). This was supplemented with a manual search of cited references from retrieved articles.

### Study selection

Studies that met the following criteria were included: (i) primary studies that determined an association between genetic polymorphism (including single nucleotide polymorphism (SNPs), deletions, duplication and copy-number variants) and cardiotoxicity; (ii) anthracycline was used and (iii) conducted in human population. Articles titles and abstracts were screened for relevancy by two independent reviewers (SWHL and SLL) and full text retrieved in accordance to the inclusion criteria. Any disagreement was resolved through adjudication with input by a third reviewer.

### Data extraction

Two reviewers (SWHL and SLL) independently extracted data from identified studies using standardised data extraction form. Reviewers compared the results and resolved any differences by discussion. Information extracted include: geographic location, ethnic group, study design, participant demographics and clinical characteristics, genotyping technique, and definition of cardiotoxicity. The study was conducted following the process specified in the PRISMA statement.

### Quality assessment

The reviewers independently assessed the quality of the included studies using quality of genetic association studies (Q-Genie) tool developed by Sohani *et al.* ^24^. This validated tool consisting of nine categories was developed based on the Strengthening the Reporting of Genetic Association Studies (STREGA)^25^ and Strengthening the Reporting of Genetic Risk Prediction Studies (GRIPS)^26^ guidelines.

### Statistical analysis

In studies which had assessed for polymorphisms of the same genotype (minimum 2 studies), we conducted a meta-analysis using a random effects model^27^. Study heterogeneity was assessed using the Cochran Q and the *I* ^2^ statistics. We also calculated the departure from Hardy-Weinberg equilibrium (HWE), which if violated, may bias the estimates and replication of postulated gene-disease associations across different studies^28^. All analyses were performed using Stata 13.0 (StataCorp, College Station, TX) and Review Manager 5.3 packages (http://comunity.cochrane.org/tools/review-production-tools/revman-5)^29^.

## Result

### Study and patient characteristics

**Figure 1:**
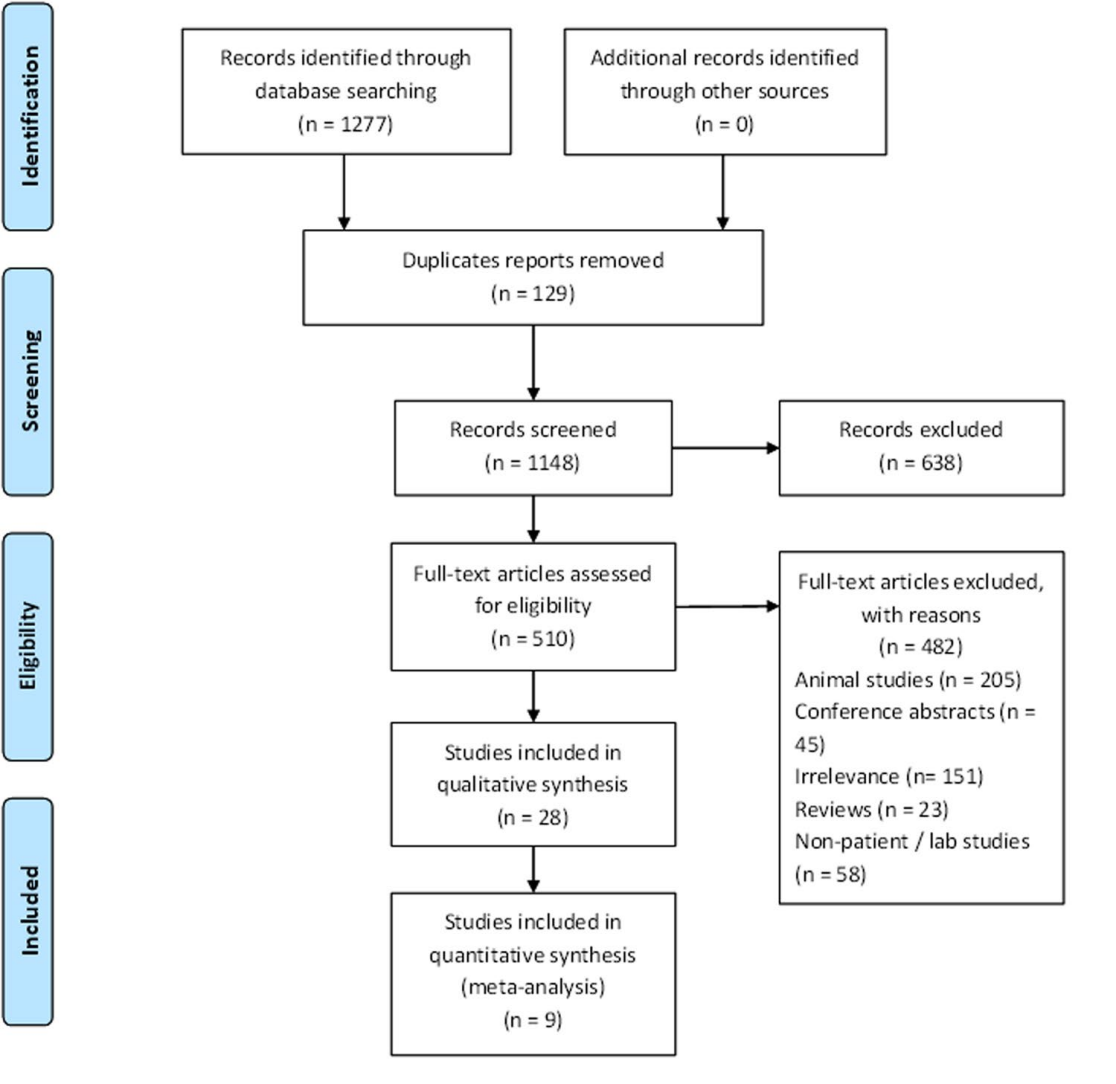
PRISMA flow diagram showing the selection process and criteria of the included studies.

Our search identified 1,277 studies and 510 underwent assessment. A total of twenty-eight studies involving 7,082 patients were included in the current review (Fig. 1). The characteristics of the included studies are presented in Table 1. Eighteen of the studies were case control studies^20–23, 30–43^, of which eight were nested case-control studies^22, 23, 32, 34–37, 42^. Another seven were prospective cohort studies^44–50^ while two were retrospective cohort study^51, 52^. The remaining one was a case report^53^. These studies were conducted in the North America (n = 16)^20, 21, 23, 30, 31, 33–37, 42–44, 46, 47, 52^, Europe (n = 9)^22, 32, 38–41, 45, 48, 51^, and Asia (n = 1)^50^ while two did not report the study location^49, 53^. Almost equal number of studies were conducted in children (n = 10) and adults (n = 13) population. Five studies included both children and adults in their report^36–39, 44^. Nineteen studies described the ethnicity of their participants^20, 23, 30–40, 43–45, 47, 50, 51^ but, there were inconsistencies in reporting of race/ethnicity. For example, Weiss *et al.* ^33^ described their participants either as Caucasian or not while Blanco *et al.* ^34^ described their participants as White, Black and others.

**Table 1:** Descriptions of included studies.

Study: Author (year)	Geographic location, ethnic group	Study design; number of participants	Age (years)		Gender: male/female		Type of Cancer examined	Anthracycline used/Cumulative dose (mg/m^2^)	Source of DNA sample	Genotyping	Definition of cardiotoxicity
			Cases	Controls	Cases	Controls					
Wojnowski (2005)^32^	Germany; 98% Germans	NCC; 550	Mean = 62.0 ± 10.9	Mean = 61.3 ± 11.0	50/37	212/151	NHL	Doxorubicin/*Cases:* Med = 504 mg IQR = 160.5 mg *Controls*: Med = 540 mg IQR = 90 mg	Peripheral blood	i) Pyrosequencing ii) RFLP	i) arrhythmia in the absence of arrhythmia before treatment ii) myocarditis-pericarditis iii) acute heart failure iv) LVEF <50% or SF <25%
Weiss(2006)^33^	USA; 85% Caucasian	CC; 197	Med = 68 (56–88)		Approx. 98/99		AML	Daunorubicin/NR	BM/peripheral blood	i) Multiplex PCR ii) Sequenom’s high-throughput matrix-assisted laser desorption/ionization time-of-flight mass spectrometry (MALDI-TOF MS)	i) SWOG toxicity criteria for SWOG 9031 ii) CTCAEv2.0 for SWOG-9333.
Blanco (2008)^34^	USA; Whites, Blacks & Others	NCC; 145	Mean = 10.3 ± 6.5	Mean = 9.1 ± 5.8	10/20	57/58	Leukaemia, brain tumour, HL, NHL, Wilms tumour, bone tumour neuroblastoma, soft tissue sarcoma,	Doxorubicin/<100 = 1 (2)* 100–350 = 13 (46) 350–500 = 7 (31)>500 = 9 (36)	Buccal cells/saliva	i) PCR-RFLP ii) Allelic discrimination with specific fluorescent probes	Self-reporting of signs and symptoms of CHF and use of medication for CHF management.
Rajic (2009)^40^	Slovenia; Caucasian	CC; 76	Mean = 25.8 ± 5.3		32/44		ALL	Not specified/Mean = 199 ± 108 Range = 24–540	Bone marrow smears	i) qPCR ii) Custom TaqMan^®^ genotyping assay	i) Clear conduction disturbances, depolarization and repolarization changes in ECG ii) SF < 30%, LVEF <54% iii) Derangement of (reference range) E (0.75 ± 0.13), A (0.51 ± 0.11), E/A (1.53 ± 0.4), IVRT (67 ± 8), PV-A (0.21 ± 0.08), PV-D (0.47 ± 0.11) PV-S (0.44 ± 0.1)
Rossi (2009)^41^	Italy; NR	CC; 106	Med = 66 (56–75)		55/51	55/51	DLBCL	Doxorubicin/15 mg/m^2^/week	Peripheral blood	SNP minisequencing	Grade 2–4 cardiotoxicity according to CTCAEv 0.3
Blanco (2012)^35^	USA; Non-Hispanic whites, Hispanics, Blacks & Others	NCC; 487	Mean = 8.3 ± 6	Mean = 8.2 ± 6	76/94	162/155	HL, NHL, bone tumours, soft tissue sarcoma, ALL, AML, other.	Not specified/*Cases:* Med = 300 (0–575) *Controls:* Med = 140 (0–1050)	Peripheral blood/buccal cells/saliva	Allelic discrimination with specific fluorescent probes	i) signs and symptoms of cardiac compromise based on American Heart Association criteria 2005 ii) Absence of symptoms/signs with echo evidence of left ventricular dysfunction (EF ≤ 40% and/or SF ≤ 28%).
Kitagawa (2012)^50^	Japan; Japanese	PC; 34	Med = 49 (21–71)		0/34		Breast cancer	Epirubicin/NR	Whole blood	TaqMan^®^ genotyping assay	i) QTc interval prolongation ii) other toxic effects based on CTCAEv3
Lubieniecka (2012)^44^	Canada; Caucasian	PC; 185	Med = 46 (14–74)		86/99		AML	Daunorubicin/NR	Blood	Sequenom genotyping assay	Percentage drops in LVEF.
Sachida-nandam (2012)^53^	NR	CS; 2	Adult		−/2		Breast cancer	Doxorubicin	Blood	PCR	NR
Semsei (2012)^51^	Hungary; Hungarian	RC; 235	Mean = 5.7 ± 3.8		126/109		ALL	Daunorubicin, doxorubicin/NR	Peripheral blood	i) Mini-sequencing ii) GenomeLab SNPstream genotyping assay	Changes in LVFS
Visscher (2012)^30^	Canada; 78% Canadian, 22% Dutch	CC; 440	Discovery Med = 5.5 (0.04–17.0) Replication Med = 6.2 (0.4–17.6) Dutch-EKZ Med = 9.0 (0.5–16.8)	Discovery Med = 3.9 (0.5–16.5) Replication Med = 3.7 (0.05–16.9) Dutch-EKZ Med = 10.6 (2.1–17.1)	Discovery = 17/21 Replication = 22/18 Dutch-EKZ = 22/21	Discovery = 66/52 Replication = 82/66 Dutch-EKZ = 27/26	ALL, AML, other leukemia, HL, NHL Osteosarcoma, Rhabdomy-osarcoma, Ewing’s sarcoma, Other sarcoma, Nephroblastoma, Hepatoblastoma, Neuroblastoma, Carcinoma	Doxorubicin, Daunorubicin/Discovery *Cases:* Med = 300 (36–540) *Controls:* Med = 175 (60–600) Replication *Cases:* Med = 270 (45–840) *Controls:* Med = 250 (25–600) Dutch-EKZ *Cases:* Med = 360 (100–720) *Controls:* Med = 300 (50–720)	NR	Custom Illumina GoldenGate SNP genotyping assay	i) SF ≤ 26% ii) sign and symptoms requiring for cardiac compromise intervention based on CTCAEv3
Volkan-Salanci (2012)^45^	Turkey; Turkish	PC; 70	Mean = 49.1 ± 13.6		7/63		Breast cancer, lymphoma, mesenchymal tumour, nasopharyngeal cancer, duodenal cancer, sarcoma	Doxorubicin, epirubicin/Mean = 317.1 ± 94.9	NR	TaqMan^®^ genotyping assay	i) LVEF decrease > 10% ii) LVEF ≤ 50%
Windsor (2012)^39^	UK, Caucasian, Afro-Caribbean, Indian/Asian	CC, 58	Med = 18 (10–51)		34/24		Osteosarcoma	Doxorubicin/NR	Peripheral blood	i) Standard PCR, ii) PRC- RFLP, iii) Multiplex PCR, iv) Illumina microarray	Decrease in LVEF by ≥ 1 CTCAEv3 grade.
Armenian (2013)^36^	USA; Non-Hispanic whites, Hispanics, Blacks & Others	NCC; 255	Med = 49.2 (16–68.8)	Med = 51.0 (6.4–72.6)	34/43	119/59	Haematology malignancy + haematopoietic cell transplant	Not specified/*Cases:* Med = 300 (60–650) *Controls:* Med = 300 (40–600)	Peripheral blood stem cells, FFPE BM core biopsies, unstained slides of BM smears	Sequenom MassARRAY	Sign and symptoms of cardiac compromise requiring intervention based American Heart Association criteria 2005
Lipshultz (2013)^46^	USA; NR	PC; 184	Med = 15.2 (3.1–31.4)		101/83		ALL	Doxorubicin/Med = 300 (204–420)	Peripheral blood	i) Pyrosequencing ii) Sequenom genotyping assay iii) TaqMan^®^ genotyping assay	i) cTnT > 0.01 ng/mL ii) NT-proBNP > 150 pg/mL (< 1 year old) iii) NT-proBNP > 100 pg/mL (≥ 1 year old)
Lubieniecka (2013)^52^	Canada; NR	RC; 91	Mean = 48.4 Range = 19–74		48/43		AML	Daunorubicin	Blood	Sequenom genotyping assay	Percentage drop in LVEF
Visscher (2013)^31^	Canada; 41% Canadian, 69% Dutch	CC; 218	Canadian-CPNDS Med = 12.6 (0.9–17.0) Dutch-EKZ Med = 9.1 (0.5–16.8)	Canadian-CPNDS Med = 4.9 (0.5–16.0) Dutch-EKZ Med = 11.2 (1.8–17.7)	Canadian-CPNDS = 8/4 Dutch-EKZ = 23/21	Canadian-CPNDS = 31/47 Dutch-EKZ = 44/40	ALL, AML, other leukemia, HL, NHL Osteosarcoma, Rhabdomyosarcoma, Ewing’s sarcoma, Other sarcoma, Nephroblastoma, Hepatoblastoma, Neuroblastoma, Carcinoma, Germ cell tumour	Doxorubicin, daunorubicin/Canadian CPDNS *Cases:* Med = 300 (175–550) *Controls:* Med = 150 (50–540) Dutch-EKZ *Cases:* Med = 360 (100–720) *Controls:* Med = 280 (50–720)	Blood/saliva/buccal swab	Custom Illumina GoldenGate SNP genotyping assay	i) SF ≤ 26% ii) sign and symptoms of cardiac compromise requiring intervention based on CTCAEv3
Vivenza (2013)^49^	NR	PC; 48	57.5 (28–73)		1/47		Breast cancer	Epirubcin/540	Blood	i) Allelic discrimination using Applera SNP assay ii) TaqMan^®^ genotyping assay	i) overt CHF (grade III) based on CTCAEv2 ii) LVEF < 50% (grade II) based on CTCAEv2
Wang (2014)^37^	USA; Non-Hispanic whites	NCC; 363	Discovery cohort Med = 19.4 (0.4–41.7)	Discovery cohort Med = 18.5 (3.5–49.2)	40/53	94/100	HL, NHL bone tumours, soft tissue sarcoma, ALL, AML, other.	Not specified/Discovery *Cases:* Med = 300 (0–630) *Controls:*Med = 152 (0–825) Replication Med = 300 (60–649)	Peripheral blood, buccal cells/saliva	Illumina IBC cardiovascular SNP array	American Heart Association criteria for cardiac compromise: i) symptoms and/or signs of cardiac compromise and echo evidence of LV dysfunction. ii) absence of symptoms/signs with echo evidence of LV dysfunction (LVEF ≤ 40% and/or SF ≤ 28%).
Wasielewski (2014)^38^	The Netherlands; Dutch	CC; 21 (Cohort I = 5; Cohort II = 13, Cohort III = 3)	Cohort I Med = 49 (2–57) Cohort II Med = 46 (34–61) Cohort III Med = 4 (4–9)		NR		Breast cancer, ALL, neuroblastoma, Wilm’s tumour, primary neuroectodermal tumour	Epirubicin, Doxorubicin, Daunorubicin/Range = 175–600	NR	Targeted next-generation DNA sequencing	i) signs and symptoms of cardiac compromise based on American Heart Association criteria (ii) echo evidence of LV dysfunction. iii) absence of symptoms/signs with echo evidence of LV dysfunction (LVEF ≤ 40% and/or SF ≤ 28%).
Aminkeng (2015)^47^	Canada; European, African, East Asia, Aboriginal Canadian	PC; Discovery = 280 Replication = 96	Discovery Med = 9.0 (2.5–14) Replication Med = 7.5 (5–12)	Discovery Med = 4.0 (2–7.5) Replication Med = 11 (6–14)	Discovery 15/17 Replication 12/10	Discovery 136/112 Replication 38/36	ALL, AML, other leukaemia, HL, NHL, osteosarcoma, rhabdomyosarcoma, Ewing’s sarcoma, other sarcoma, hepatoblastoma, neuroblastoma, Wilms tumour	Doxorubicin, Daunorubicin, Epirubicin/Discovery *Cases:* Med = 260 (177.5–365) *Controls:* Med = 175 (140–295) Replication *Cases:* Med = 407.5 (270–480) *Controls:* Med = 277.5 (180–364)	NR	Illumina HumanOmniExp-ress assay	i) LVEF < 45% ii) Dilation of LV-end-diastolic dimension >117%.
Krajinovic (2015)^43^	Canada, French-Canadian	CC; 295	QcALL cohort Mean = 6.16 DFCI cohort Mean = 5.27		QcALL cohort = 134/117 DFCI cohort = 21/23		ALL	Doxorubicin/300–360	Blood, buccal swabs	PCR allele-specific-oligonucleotide hybridization assays.	Reduction in SF and EF
Reichwagen (2015)^22^	Germany, Czech Republic & Switzerland; NR	NCC; 520	Med = 68(61–80)	Med = 67(62–79)	25/31	46/48	NHL	Doxorubicin/*Cases:* Med = 309 *Controls:* Med = 318	Blood	i) Pyrosequencing ii) TaqMan® genotyping assays	Grade >0 based on CTCAEv2
Visscher (2015)^21^	Canada & The Netherlands; NR	CC; 536	Med = 7.4(0.04–17.6)	Med = 4.9(0.1–17.7)	64/58	211/187	Leukaemia, lymphoma, sarcoma, blastoma and others	Doxorubicin, Daunorubicin/*Cases:* Med = 300 (36–840) *Controls:* Med = 200 (25–740)	Blood, saliva, buccal swabs	Custom Illumina GoldenGate SNP genotyping assay	i) Shortening fractions <26% ii) Echo and/or symptoms of cardiac compromise requiring intervention based on CTCAEv3
Vulsteke (2015)^48^	Belgium; NR	PC; 877	Mean = 50.3		NR		Breast cancer	Epirubicin/NR	Blood	Sequenom MassARRAY	(ii)asymptomatic decrease of LVEF>10%
Hertz (2016)^20^	USA; White, Black, Other	CC, 166	Med = 50 (35–64)	Med = 50 (24–80)	0/19	0/147	Breast cancer	Doxorubicin/*Cases:* Med = 240 (240–350) *Controls:* Med = 240 (120–366)	Blood	i) Sequenom MassARRAY ii) TaqMan^®^ allelic discrimination assay	EF<55%
Reinbolt (2016)^42^	USA; NR	NCC, 162	Mean = 51.9 ± 11.9	Mean = 50.1 ± 9.3	0/52	0/110	Breast cancer	NR	NR	i) TaqMan^®^ allelic discrimination assay ii)	i) EF <50% ii) decrease of LVEF>15% iii) new arrhythmia iv) new myocardial infarction
Wang (2016)^23^	USA; Non-Hispanic white, Hispanic, others	NCC; 385 (Discovery = 331, Replication = 54)	Discovery Set Mean = 8.4 ± 5.7 Med = 7.5 (0–20) ReplicationSet Mean = 7.7 ± 5.0 Med = 7.7 (0.02–20.6)	Discovery Set Mean = 8.3 ± 5.8 Med = 7.9 (0–21)	Discovery Set: 46/66 Replication Set: 30/24	Discovery Set:106/113	HL, NHL, Sarcoma, AML, ALLand others	NR/Discovery *Cases:* Med = 319 (0–760) *Controls:* Med = 180 (0–825) Replication *Cases:* Med = 350 (0–668) *Controls:* Med = 301 (0–668)	Blood, buccal cells, saliva	i) Illumina HumanOmniExp-ress assay ii) Sequenom MassARRAY	i) signs and symptoms of cardiac compromise based on American Heart Association criteria 2009 ii) absence of symptoms/signs with echo evidence of LV dysfunction (LVEF ≤ 40% and/or SF ≤ 28%).

ALL, acute lymphoblastic leukaemia; AML, acute myeloid leukaemia, BM, bone marrow; CC, case-control; EF, ejection fraction; FFPE, Formalin-fixed, paraffin-embedded; HL, Hodgkin’s lymphoma; LVEF, left ventricular ejection fraction; LVFS, left ventricular shortening fraction; Med, median; CTCAE, National Cancer Institute Common Toxicity Criteria; NCC, nested case control; NHL, non-Hodgkin’s lymphoma, NR, not reported, PC, prospective cohort; RC, retrospective cohort; RFLP, restriction fragment length polymorphism; SF, shortening fraction.

The most common type of cancer examined were leukaemia (n = 7), breast cancer (n = 6), lymphoma (n = 3) and osteosarcoma (n = 1). In the other eleven studies, the authors examined a mix types of cancer. Doxorubicin (n = 8), daunorubicin (n = 4) and epirubicin (n = 3) were the common anthracyclines examined. Only eight studies reported the cumulative anthracycline dose in doxorubicin isotoxic equivalent doses^21, 23, 30, 31, 35–37, 47^. The median cumulative doses in doxorubicin isotoxic equivalent dose ranged from 240 to 504 mg/m^2^ for cases and 175 to 540 mg/m^2^ for controls. These conversions were mainly derived based upon the guidelines of the Children’s Oncology Group^54, 55^.

The definition of cardiotoxicity varied across studies, with most studies using either a subjective outcome (n = 5), objective outcome (n = 8) or both (n = 14) while one study did not define cardiotoxicity^53^ (Supplementary Table 1). Most studies using subjective outcomes defined cardiotoxicity as the presence of signs and symptoms requiring intervention^21–23, 30, 31, 33, 35–37, 41, 47–49^. In addition, some studies have used the left ventricular ejection fraction (LVEF) or shortening fraction (SF) as an objective measure, but the cut-off points varies. For example, the cut-off values of less than 40% to 55% of LVEF^20^ or decrease of more than 10–15% have been used. Three studies also included electrocardiogram changes in the definition of cardiotoxicity i.e. arrhythmia^22, 32, 42^ and abnormalities in ECG^40^ while one study solely examined the effect of anthracycline on QT interval and arrhythmia^50^.

Blood and buccal cells were the most common bio-specimen used for genotyping. Fifteen studies used single bio-specimen of either, blood^20, 22, 32, 39, 41, 44, 46, 48–53^, buccal swab^34^ or bone marrow smear^40^ while seven studies used more than one type of bio-specimens^21, 23, 31, 33, 35–37^. Six studies did not report the bio-specimen used for genotyping^30, 38, 42, 43, 45, 47^. Seventeen studies used single genotyping assay^21, 30, 31, 35–38, 41–45, 47, 48, 50, 52, 53^ while the remaining eleven studies use multiple genotyping assays^20, 22, 23, 32–34, 39, 40, 46, 49, 51^. The most commonly used assay technique were TaqMan® genotyping assay (n = 7), Sequenom MassARRAY (n = 4), Sequenom genotyping assay (n = 3), custom Illumina GoldenGate SNP genotyping assay (n = 3) and pyrosequencing (n = 3). Twenty-one studies assessed their cohort or control group for compliance with the HWE^20–23, 30–32, 34–39, 41, 44, 45, 47–49, 51, 52^.

### The quality of the reporting in the studies

Among the reviewed studies, twenty-six studies were rated to have high quality (mean score of 45 for studies with control group and 40 for studies without control group) except for one study^44^, which was rated to be of moderate quality (Supplementary Table 2). On average, included studies were rated as good for most of the items on the Q-Genie tool except for the domain: sample size and power as studies had not described or determined the sample size required for their studies. In most cases, these were either retrospectively analyses of a research datasets/cohort assembled for different purposes.

### Anthracycline-induced cardiotoxicity and genotype

**Figure 2:**
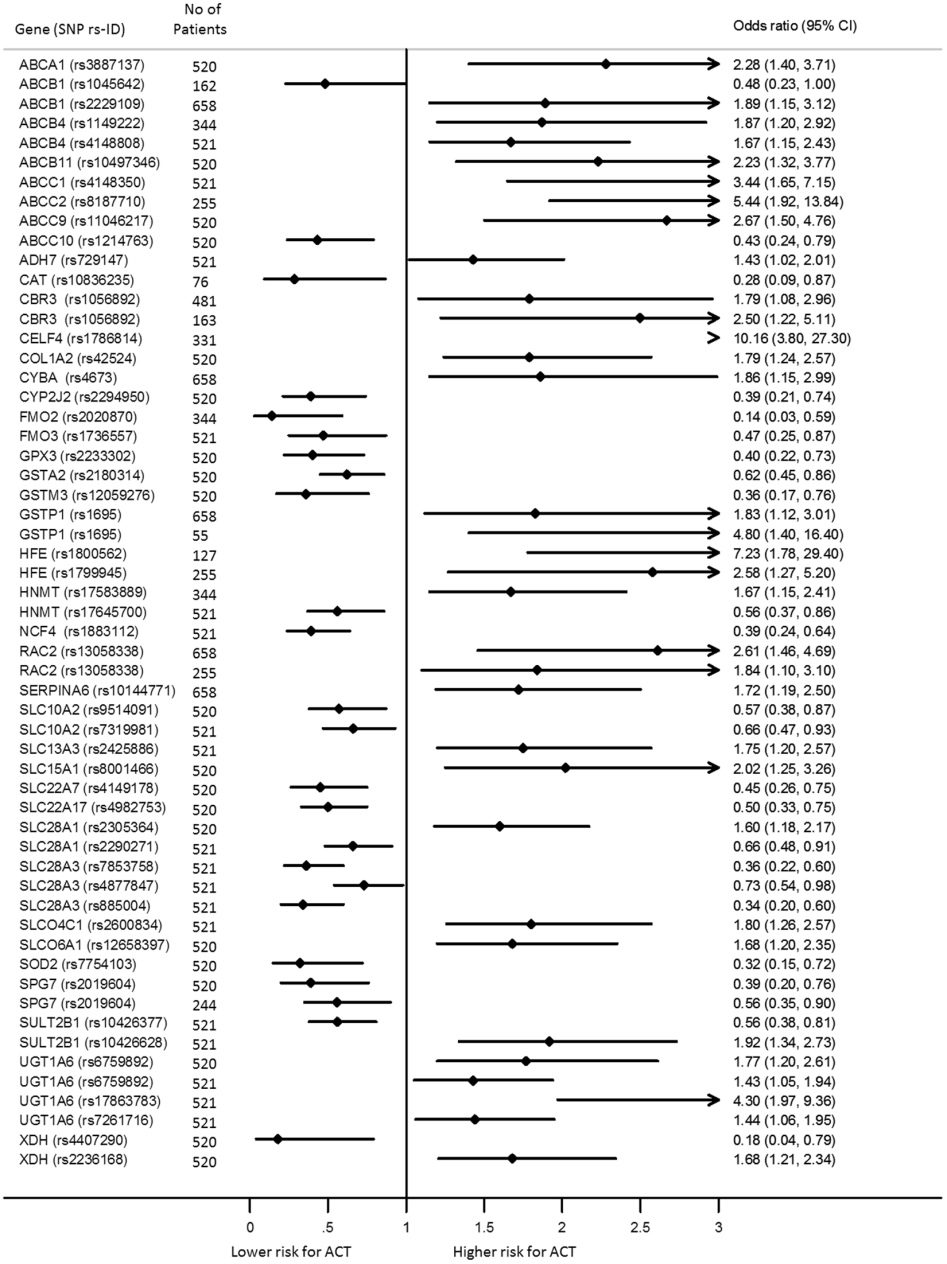
Forest plot of SNPs which examined the association of developing anthracycline-induced cardiotoxicity. SNPs significantly associated with ACT with no odds ratio or confidence interval reported are ABCC1 (rs3743527, rs246221, rs45511401), ABCC5 (rs7627754), AKR1C4 (rs7083869, rs2151896), CBR3 (rs10483032), CYP1A2 (rs2069522, rs2069526, rs4646427), CYP2B6 (rs7255904, rs1709115), CYP4B1 (rs837400, rs4646495), CYP4F11 (rs8112732, rs12610962, rs2072270), HSD17B2 (rs16956248, rs13333826, rs7196087, rs2955159, rs2966245), HSD17B4 (rs257970, rs2636968), KCNH2 (rs3807375), POR (rs2868177, rs13240755, rs4732513), SLC22A17 (rs11625724, rs12882406, rs12896494). The diamond in each line represents the effect estimate and weight of each study. The width of the line across the diamond shows the 95% confidence interval of the effect estimate of individual studies. ACT, Anthracycline-induced cardiotoxicity; CI, confidence interval.

**Figure 3:**
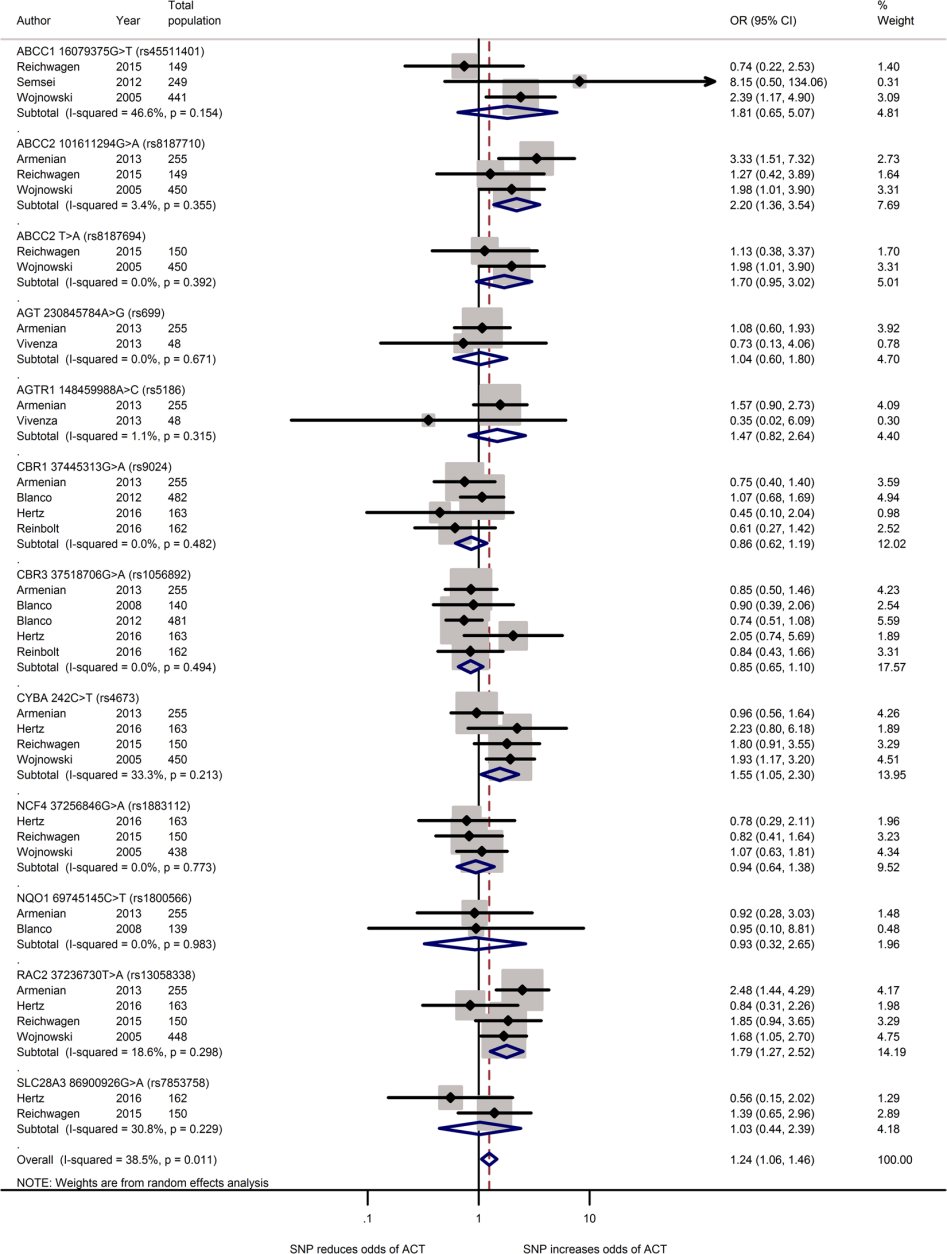
Forest plot of meta-analysis for 12 SNPs. Three variants, ABCC2 rs8187710, CYBA rs4673 and RAC2 rs13058338, are significantly increased the odds for ACT.

A total of 147 SNPs involving eighty-four genes were reported by the studies (Supplementary Table 3). Three genome-wide association studies^23, 39, 47^ were identified, and the remaining studies involved using a candidate gene approach. Most of the studies focused on variation in genes implicated in biosynthesis of anthracyclines or cardiac function. Eighty-seven of the SNPs were reported to be significantly associated with ACT by at least one study (Fig. 2). Quantitative analysis was possible for twelve polymorphs in eleven genes (Fig. 3). Most of the SNPs were from genes which encode transporter proteins; of which twenty-eight SNPs were from eleven ATP-binding cassette (ABC) transporters gene while nineteen SNPs were eleven genes encode solute carriers (SLC). The most studied genes encoding metabolising proteins were genes encode aldo/keto reductase (AKR) superfamily and carbonyl reductase (CBR). A discussion on the genes included in meta-analysis follows below.

#### ATP Binding Cassette (ABC) gene

ABC transporters genes encode a superfamily of transmembrane proteins that actively transport substrates including doxorubicin across membranes using adenosine triphosphate^56^. Fourteen of the twenty-eight variants in ABC transporters were found to significantly increase the risk for ACT^20, 21, 30–32, 36, 41, 43, 48, 51^ (Supplementary Table 3). ABCC1 is the most studied gene with nine SNPs followed by ABCB1 (5 SNPs) and ABCC2 (3 SNPs). The rs246221 polymorphism of ABCC1 gene was found to significantly deteriorate cardiac function in both studies^48, 51^. Seven SNPs, rs1045642^20, 41^, rs1149222^20, 30, 31, 53^, rs4148808^20, 31^, rs45511401^22, 32, 51^, rs4148350^20, 30^, rs8187710^22, 32, 36^ and rs8187694^22, 30, 32^ were found to increase the risk in only one of the studies assessing their association with ACT.

Armenian *et al.* recruited 77 cases and 178 controls from a population of haematological patients that underwent haematopoietic cell transplantation reported that rs8187710 increased ACT risk (OR: 5.22; 95% CI: 1.92–13.84; false discovery rate-adjusted p = 0.02)^36^. Using similar study design and a larger sample size (87 cases and 363 controls) of only non-Hodgkin lymphoma survivors, Wojnowski *et al.* reported the heterozygous or homozygous genotypes risk of acute ACT was statistically significant (OR: 2.3; 95% CI: 1.0–5.4; Fisher exact test p = 0.06)^32^. In contrast, Reichewagen *et al.* did not find significant association between the mutation and risk for ACT (OR: 1.3; 95% CI: 0.4–3.9; p = 0.67)^22^. When combined, the missense mutation was associated with a large increase in risk (pooled OR: 2.20; 95% CI: 1.36–3.54; p = 0.001).

Meta-analysis of three studies in European^22, 32, 51^ populations revealed that the missense mutation of rs45511401 increased the risk for ACT (pooled OR: 1.81; 95% CI: 0.65–5.07; p = 0.26) with moderate heterogeneity (*I* ^2^ = 47%). Similarly the combined effect of ABCC2 rs8187694 from two studies in European^22, 32^ populations showed no significant association (pooled OR: 1.70; 95% CI: 0.95–3.02; p = 0.07).

#### Carbonyl reductases (CBR) gene

Carbonyl reductases (CBR) genes encode enzymes that catalyse the reduction of endogenous aliphatic aldehydes and ketones and various xenobiotic, thus offering cardio-protective role against ACT. Four SNPs on carbonyl reductases (CBR) were studied, one on carbonyl reductase 1 gene (CBR1) and three on carbonyl reductase 3 gene (CBR3). However, two SNPs, rs9024 of CBR1 and rs1056892 of CBR3 were associated with cardio-protection, but this did not reach statistical significance (pooled OR: 0.86; 95% CI: 0.62–1.19 and 0.85; 0.65–1.10 respectively, Fig. 3).

#### Cytochrome b-245, alpha polypeptide (CYBA) gene

Cytochrome B-245, alpha polypeptide gene (CYBA, NC_000016.10) encodes the primary component of the microbicidal oxidase system of phagocytes. We identified six studies which assessed associations of the rs4673 missense SNP of CYBA with ACT, three studies^20, 22, 32^ are included in qualitative analysis due to unavailability of required information in the other two studies^30, 41^. Among the samples, the SNP was found to increase the odds of developing ACT (pooled OR: 1.55; 95% CI: 1.05–2.30; p = 0.03) with moderate heterogeneity (*I* ^2^ = 33%).

#### Neutrophil cytosolic factor 4 (NCF4) gene

Neutrophil cytosolic factor 4 gene (NCF4, NC_00002.10) encodes the p40phox subunit of the NAD(P)H oxidase^57^. The rs1883112 polymorphism at the putative promoter of NCF4 blocks oxidase activation of the enzyme thus reduces the formation of reactive oxidant intermediates^58^. Two of the six studies examined the effect of SNP rs1883112 found that SNP was significantly associated with cardiac toxicity^32, 36^. The combined effect of this synonymous substitution from two studies in North America^20, 36^ and European^22, 32^ populations showed no significant association (pooled OR: 0.94; 95% CI: 0.64–1.38; p = 0.75).

#### Ras-Related C3 Botulinum Toxin Substrate 2 (RAC2) gene

Ras-Related C3 Botulinum Toxin Substrate 2 gene (RAC2, NC_000022.11) encodes the protein regulating diverse processes including secretion, phagocytosis, cell polarisation and generation of reactive oxygen species. Three of six studies reported SNP rs13058338 on RAC2 significantly increase risk for ACT^32, 36, 41^. Analysis of this intron variant in four studies showed that RAC mutation increased the risk of cardiotoxicity by nearly two times (pooled OR: 1.79; 95% CI: 1.27–2.52; p < 0.001).

## Discussion

**Figure 4:**
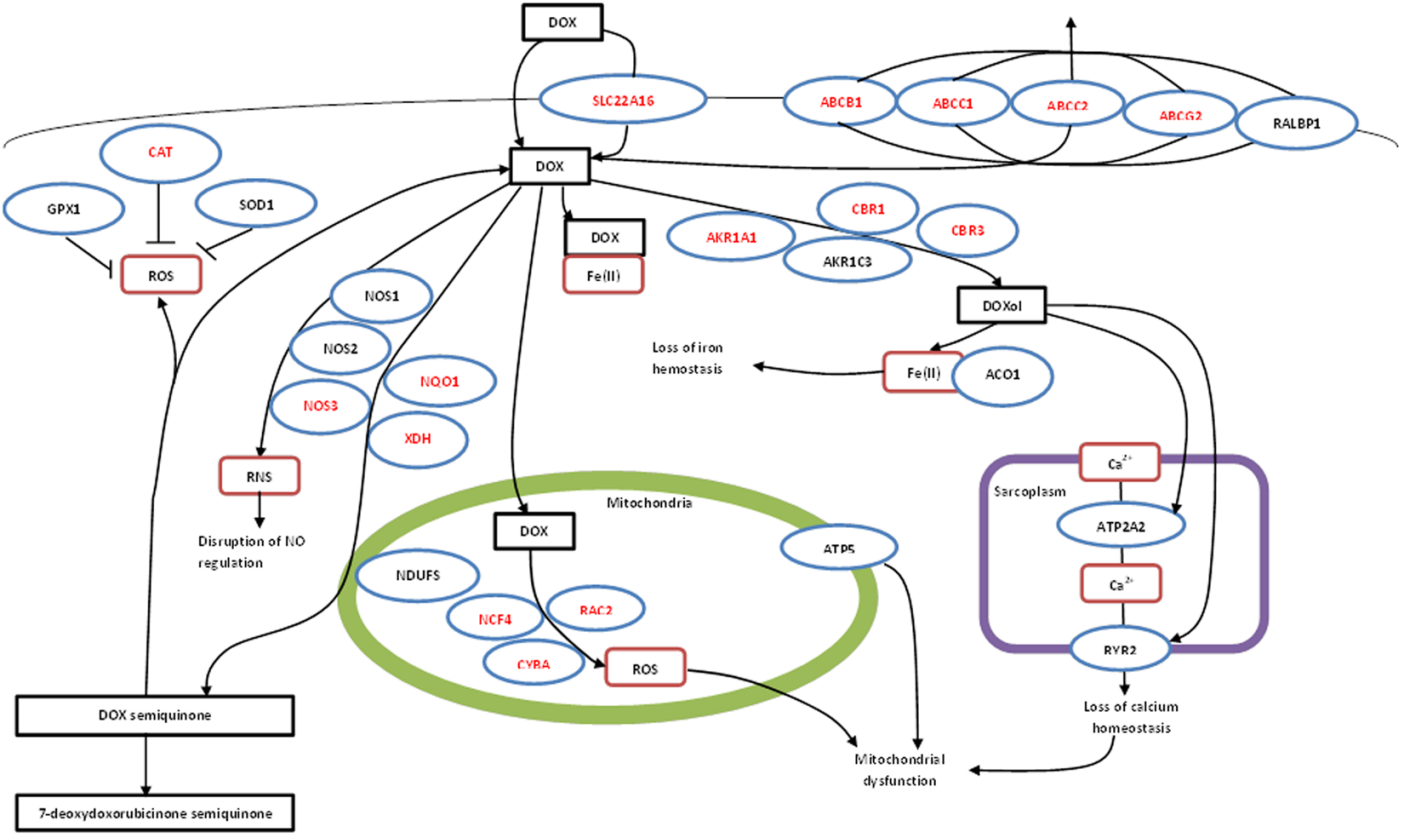
Diagrammatic representative of the candidate genes involved in transport and metabolism of doxorubicin and doxorubicin induced cardiotoxicity. ABCB1, ATP-Binding Cassette Subfamily B Member 1; ABCC1, ATP-Binding Cassette Subfamily C Member 1; ABCC2, ATP-Binding Cassette Subfamily C Member 2; ABCG2, ATP-Binding Cassette Subfamily G Member 2, ACO1, Aconitase 1; AKR1A1, Aldo-Keto Reductase Family 1 Member A1, AKR1C3, Aldo-Keto Reductase Family 1 Member C3; ATP2A2, ATPase Sarcoplasmic/Endoplasmic Reticulum Ca^2+^ Transporting 2; ATP5E, ATP synthase H^+^ Transporting, mitochondrial F1 Complex, Epsilum Subunit; CAT, Catalase gene; CBR1, Carbonyl Reductase 1; CBR3, Carbonyl Reductase 3; CYBA, Cytochrome B-245 Alpha Chain; GPX1, Glutathione Peroxidase 1; NCF4, Neutrophil Cytosolic Factor 4; NDUFS, NADH: Ubiquinone Oxidoreductase Subunit; NOS1, Nitric Oxide Synthase 1; NOS2, Nitric Oxide Synthase 2; NOS3, Nitric Oxide Synthase 3; NQO1, NAD(P)H Quinone Dehydrogenase 1; RAC2, Ras-related C3 Botulinum Toxin Substrate 2; RALBP1, RalA Binding Protein 1; RYR2, Ryanodine Receptor 2; SLC22A16, Solute Carrier Family 22 Member 16; SOD1, Superoxide Dismutase 2, mitochondrial; XDH, Xanthine Dehydrogenase.

To our knowledge, this is the first and only systematic review which examined the role of genetic polymorphisms with ACT induced cardiotoxicity. We found a total of twenty-eight studies, examining eighty-four different genes. Most of the genes studied were linked to the biochemical pathway of anthracycline, oxidative stress or cardiac function (Fig. 4). As such, it is not surprising that all but one^47^ genetic studies described in this article have included these candidate genes in their study. Results from our meta-analyses revealed that polymorphism in three (3.6%) of the eight-four genes were significantly associated with an increased odds of cardiotoxicity in individuals treated with anthracyclines. However, the individual risk provided by any of these candidate genes were moderate only (OR: 1.55–2.20), in agreement with previous studies which have examined other complex diseases, such as stroke^59^ and ischaemic heart diseases^60, 61^.

For the genes that were found to have a positive association, animal and mechanistic studies have shown that these alleles alter the expression or activity of the encoded protein and thus contribute to disease pathogenesis. ABCC2 gene encodes for proteins that are involved the efflux of substances from cells, and mutation of ABCC2 significantly reduces ATPase activity, resulting in a decrease in efflux activity leading to intracellular accumulation of anthracycline^62^. Similarly, the Rac2 (Ras-related C3 botulinum toxin substrate 2) encoded by RAC2 gene is a mitochondrial protein that is required in electron transfer reaction of NADPH oxidase^63^ during the formation of reactive oxygen species (ROS)^64^. Alteration of the gene results in mitochondrial dysfunction and thus an increase ROS production, which ultimately leads to myocytes damages. Taken together, mutations in these genes are thought to result in cardiomyopathy due to accumulation of anthracycline and excessive ROS in myocytes.

We also observed that some of these genes were not only related to cardiotoxicity, but also other adverse drug reactions (ADRs) of chemotherapy such as myelosuppression and infection as well as overall survival. The SNPs ABCG2 rs2231142^41^, NCF4 rs1883112^41^, GSTP1 rs1695^39, 41^, CYBA rs4673^39^ and GSTM1 null allele^39^ significantly increased odds for grade 3–4 hematologic toxicity in patients treated with anthracycline-based chemotherapy regimen. Similarly, ABCB1 rs1045642, ABCG2 rs2231137 and NCF4 rs1883112 significantly increased odds for grade 2–4 infection^41^. In addition, rs1695 of GSTP1^39^, rs17222723 of ABCC2^41^ and rs4673 of CYBA^41^ were significantly related to progression-free survival or event-free survival.

This study has some limitations which warrant discussion. Firstly, we found a total of 147 SNPs which were examined for the possible association with ACT. Most of the SNPs have only been examined once; which limited our ability to perform a meta-analysis. In addition, there were inconsistencies in reporting of results between studies. As such, our meta-analyses only included between two to five studies, which restricted subgroup analyses. The included studies were also heterogeneous and had not adjusted for confounders, which further limits the precision of overall estimates. We also selectively discussed the roles of genes included in the meta-analysis. It should be noted that the SNPs discussed in this review does not imply that they are superior in any aspect to other SNPs identified. Many of the studies were not prospectively designed but had used a convenience sampling, which is reinforced by the fact that none of the studies had adequately reported the sample size calculations. Similarly, nearly all of the studies (96%) of the studies were carried out in Western populations, thus limiting the generalisability to other populations. Furthermore, most of the studies had not reported the demographics of their population. Finally, only a handful studies had adjusted for some confounding factors in their analysis, although these have been shown to increase the risk factor for AIC.

Over the past few decades, the development in molecular biology has increased our understanding on the role of genetic variation underlying adverse drug reactions (ADRs). Currently, genetic testing is recommended for identifying patients at risk for ADRs. Examples include testing of thiopurine methyltansferase (TMPT) gene variation prior to thiopurine therapy in inflammatory bowel disease and human leukocyte antigen (HLA)-B*1502 for treatment of seizures with carbamazepine. Polymorphisms of TMPT gene have been known to cause lowered TPMT activity, and thus a reduced dose is recommended for heterozygous patients to prevent hematopoietic toxicity^65^. Meanwhile, HLA-B*15:02 screening is recommended for Asian populations to identify patients at risk for carbamazepine-induced Stevens-Johnson syndrome and toxic epidermal necrolysis^66^.

However, results from this study suggest that unlike examples listed above, several polymorphs may be involved in ACT. As such, a genome-wide association studies which could examine SNPs across the whole genome should be conducted. In order to ensure that study findings can be more effective to influence the development of personalised medicine for addressing drug toxicities in general and ACT in specific, future studies should ideally be conducted in a prospective large cohort. Multicentre studies including patients from other continents especially Africa, Asia, South America, Australia and Oceania, are encouraged. In addition, the use of an objective definition of cardiotoxicity and reporting the frequency of events for each genotype should be considered.

## Conclusions

Results of this study indicate that several polymorphisms of pharmacogenetics candidates across the anthracyclines biochemistry and cardiomyopathy pathways are potentially a predictor for ACT. However, the evidences are limited and too heterogeneous for a significant quantitative analysis. Further studies are needed to generate robust genetic predictor(s) for ACT to achieve the goal of individualising anthracycline therapy.

## Electronic supplementary material

Supplementary Information
