# Supplementary material for: Candidate Gene Association Studies of Anthracycline-induced Cardiotoxicity: A Systematic Review and Meta-analysis
Source: Sci Rep. 2017 Feb 27;7:39. doi: 10.1038/s41598-017-00075-1 (PMC5428315; doi:10.1038/s41598-017-00075-1)
Supplement: Supplementary file 1 — Supplementary Information [file 41598_2017_75_MOESM1_ESM.pdf]

Title Page

Title: Candidate Gene Association Studies of Anthracycline-induced Cardiotoxicity: A Systematic Review and Meta-analysis.

Authors: Siew Lian Leong[1,2], Nathorn Chaiyakunapruk[1,3,4,5], Shaun Wen Huey Lee [1*]

Supplementary Information

Search Strategies
Complete list of search terms used.

Supplementary Table 1: Definition of cardiotoxicity used by included studies. *This table summarised the various definition for cardiotoxicity used in the included studies.* (Words)

Supplementary Table 2: The quality assessment of reporting in each study (N = 27). *Each study was scored independently by 2 reviewers according the 11 items in Q-Genie tool. The average score of each item and total average score for each study were summarised in this table. (Words)*

Supplementary Table 3: Genetic polymorphisms, association with ACT and HWE status. *This table summarised the findings on the association between each of the 147 SNPs in 84 genes and anthracycline-induced cardiotoxicity.*
(Words)

Search Strategies

1. OVID Medline, Cochrane Central Register of Controlled Studies, EMBASE and AMED Text word search

(1) anthracycline*.mp.; (2) doxorubixin*.mp.; (3) daunorubicin*.mp. (4) epirubicin*.mp. (5) idarubicin*.mp. (6) 1 or 2 or 3 or 4 or 5; (7) cardiotoxicity*.mp.; (8) cardiomyopathy*mp.; (9) heart*.mp.; (10) failure*.mp.; (11) 9 and 10; (12) arrhythmia*.mp.; (13) 7 or 8 or 11 or 12; (14) genetic*.mp.; (15) polymorphism*.mp.; (16) pharmacogenomics*.mp.; (17) variant*.mp. (18) 14 or 15 or 16 or 17; (19) 6 and 13 and 18

2. PUBMED Text word Search

(1) anthracycline or doxorubicin or daunorubicin or epirubicin or idarubicin; (2) heart and failure; (3) 2 or cardiotoxicity or cardiomyopathy or arrhythmia; (4) genetic or pharmacogenomics or variant or polymorphism (5) 1 and 3 and 4

3. CINAHL Plus Text word search

(1) anthracycline or doxorubicin or daunorubicin or epirubicin or idarubicin; (2) cardiotoxicity or cardiomyopathy or arrhythmia or heart failure; (3) genetic or pharmacogenomics or variant or polymorphism; (4) 1 and 2 and 3

4. HuGE Navigator Text word search

anthracycline and cardiotoxicity and genetic

Supplementary Table 1: Definition of cardiotoxicity used by included studies.

Studies	Definition
Wojnowski, 2005	arrhythmia in the absence of arrhythmia before treatment *or* myocarditis-pericarditis *or* acute heart failure *or* LVEF <50% or SF <25%
Weiss, 2006	SWOG toxicity criteria for SWOG 9031 *or* CTCAEv2.0 for SWOG-9333.
Blanco, 2008	Self-reporting of signs and symptoms of CHF and use of medication for CHF management.
Rajic, 2009	Clear conduction disturbances, depolarization and repolarization changes in ECG *or* SF < 30%, *or* LVEF <54% *or d*erangement of (reference range) E (0.75±0.13), A (0.51±0.11), E/A (1.53±0.4), IVRT (67±8), PV-A (0.21±0.08), PV-D (0.47±0.11) PV-S (0.44±0.1)
Rossi, 2009	Grade 2-4 cardiotoxicity according to CTCAEv 0.3
Blanco, 2012	Signs and symptoms of cardiac compromise based on AHA criteria 2005 *or* absence of symptoms/signs with echo evidence of left ventricular dysfunction (EF ≤ 40% and/or SF ≤ 28%).
Kitagawa, 2012	QTc interval prolongation *or* other toxic effects based on CTCAEv3
Lubieniecka, 2012	Percentage drops in LVEF.
Semsei, 2012	Changes in LVFS
Visscher, 2012	SF ≤ 26% *or* sign and symptoms requiring for cardiac compromise intervention based on CTCAEv3
Volkan-Salanci, 2012	LVEF decrease > 10% *or* LVEF ≤ 50%
Windsor, 2012	Decrease in LVEF by ≥ 1 CTCAEv3 grade.
Armenian, 2013	Sign and symptoms of cardiac compromise requiring intervention based AHA criteria 2005
Lipshultz, 2013	cTnT > 0.01 ng/mL or NT-proBNP > 150 pg/mL (< 1 year old) or NT-proBNP > 100 pg/mL (≥ 1 year old)
Lubieniecka, 2013	Percentage drop in LVEF
Visscher, 2013	SF ≤ 26% *or* sign and symptoms of cardiac compromise requiring intervention based on CTCAEv3
Vivenza, 2013	Overt CHF (grade III) based on CTCAEv2 *or* LVEF < 50% (grade II) based on CTCAEv2
Wang, 2014	AHA criteria for cardiac compromise i.e. symptoms and/or signs of cardiac compromise and echo evidence of LV dysfunction *or* absence of symptoms/signs with echo evidence of LV dysfunction (LVEF ≤ 40% and/or SF ≤ 28%).
Wasielewski, 2014	Signs and symptoms of cardiac compromise based AHA criteria *or* echo evidence of LV dysfunction *or* absence of symptoms/signs with echo evidence of LV dysfunction (LVEF ≤ 40% and/or SF ≤ 28%).
Aminkeng, 2015	LVEF < 45% *or d*ilation of LV-end-diastolic dimension >117%.
Krajinovic, 2015	Reduction in SF and EF
Reichwagen, 2015	Grade >0 based on CTCAEv2
Visscher, 2015	SF <26% *or e*cho and/or symptoms of cardiac compromise requiring intervention based on CTCAEv3
Vulsteke, 2015	Asymptomatic decrease of LVEF>10%
Hertz, 2016	EF<55%
Reinbolt, 2016	EF <50% *or* decrease of LVEF>15% *or* new arrhythmia *or* new myocardial infarction
Wang, 2016	Signs and symptoms of cardiac compromise based on AHA criteria 2009 *or* absence of symptoms/signs with echo evidence of LV dysfunction (LVEF ≤ 40% and/or SF ≤ 28%).

Sachidanandam, 2012 did not report the definition of cardiotoxicity used.
Abbreviation: AHA, American Heart Association; CHF, congestive heart failure; ECG, electrocardiogram; EF, ejection fraction; LV, left ventricular; LVEF, left ventricular ejection fraction; LVFS, left ventricular shortening fraction; CTCAE, National Cancer Institute Common Toxicity Criteria; SF, shortening fraction; SWOG, Southwest Oncology Group

Supplementary Table 2: The quality assessment of reporting in each study (N = 27[1]).

Item \ Study	1	2	3	4	5	6	7	8	9	10	11	12	13	14
**A**	6	5.5	5	7	7	6	6	6	6.5	4.5	6	6	6	6
**B**	5.5	5	5	7	6	7	6	5	5.5	5	5	6	6	5
**C**	5.5	4	5.5	5	3	6.5	0	0	0	5	0	4	7	5
**D**	5	5	5	6	6	5	6	5	5.5	4.5	4.5	6	5	5.5
**E**	5	4.5	4.5	2	4	4.5	4	3	5	3.5	3.5	7	4.5	3.5
**F**	4	3	4	2	5	4	3.5	3.5	4	3.5	3.5	6	5	3.5
**G**	3	3.5	2.5	1	7	3	2.5	3	3	3	2.5	1	2.5	6
**H**	5	4.5	4.5	4	7	5	4	3	5.5	5	5	6	5	6
**I**	6	4.5	5.5	4	7	6	3	2.5	6	4.5	5	6	5	5
**J**	3.5	4	4	5	4	4	3.5	3	3.5	4	3.5	6	4	4
**K**	6	5	4	5	6	6	4	4.5	5.5	5	5	6	5	4
**Total scoring**	54.5	48.5	49.5	48	62	57	42.5	38.5	50	47.5	43.5	60	55	53.5
**Quality[1]**	G	G	G	G	G	G	G	M	G	G	G	G	G	G

Reported rate is an average rate given by two investigators. [1]Study by Sachinandam, 2012 is not rated because it is a case study

1, Wojnowski, 2005; 2, Weiss, 2006; 3, Blanco, 2008; 4, Rajic, 2009; 5, Rossi, 2009; 6, Blanco, 2012; 7, Kitagawa, 2012; 8, Lubieniecka, 2012; 9, Semsei, 2012; 10, Visscher, 2012; 11, Volkan-Salanci, 2012; 12, Windsor, 2012; 13, Armenian, 2013; 14, Lipshultz, 2013; 15, Lubieniecka, 2013; 16, Visscher, 2013; 17, Vivenza, 2013; 18, Wang, 2014; 19, Wasielewski, 2014; 20, Aminkeng,2015; 21, Krajinovic, 2015; 22, Reichwagen, 2015; 23, Visscher, 2015; 24, Vulsteke, 2015; 25, Hertz, 2016; 26, Reinbolt, 2016; 27, Wang, 2016.

A, Rationale of Study; B, Selection and definition of outcome; C, Selection and comparability of comparison groups (0 if not applicable); D, Technical classification of the exposure; E, Non-technical classification of the exposure; F, Other source of bias; G, Sample size and power; H, A priopri planning of analyses; I, Statistical methods and control for confounding; J, Testing of assumption & inferences for genetic analyses; K, Appropriateness of inferences drawn from results. [1]G = Study of good quality, M =Study of moderate quality.

Supplementary Table 2: The quality assessment of reporting in each study (N = 27[1]). *(continued)*

Item \ Study	15	16	17	18	19	20	21	22	23	24	25	26	27
**A**	6	5.5	6	5	6.5	5.5	6	6	6	5.5	6	6	6
**B**	6	6	6.5	5	7	5	5.5	6.5	6.5	5	6	6	6
**C**	0	5.5	6	0	7	5.5	0	6	6	5.5	5	7	7
**D**	6	5	6	3.5	6	4.5	6	6	6	4.5	6	6	6
**E**	5	4	4	4	7	4.5	3	2	2	4.5	4	6	6
**F**	5	3.5	5	4.5	6.5	4.5	5	5.5	5.5	4.5	5	4	5
**G**	3	2	1	2.5	1	1	3	1	1	1	4	1	7
**H**	5	5	5	4.5	7	5	5	6.5	6.5	5	6	5	6
**I**	6	6	5.5	5	6.5	5.5	5.5	6	6	5.5	4	6	7
**J**	5	4.5	3	3.5	6	4.5	5	5.5	5.5	4.5	6	5	6
**K**	5	5	6.5	4	6.5	6.5	6	6	6	6.5	6	6	6
**Total scoring**	52	52	54.5	41.5	67	52	50	57	57	52	58	58	68
**Quality***	G	G	G	G	G	G	G	G	G	G	G	G	G

Reported rate is an average rate given by two investigators. [1]Study by Sachinandam, 2012 is not rated because it is a case study

1, Wojnowski, 2005; 2, Weiss, 2006; 3, Blanco, 2008; 4, Rajic, 2009; 5, Rossi, 2009; 6, Blanco, 2012; 7, Kitagawa, 2012; 8, Lubieniecka, 2012; 9, Semsei, 2012; 10, Visscher, 2012; 11, Volkan-Salanci, 2012; 12, Windsor, 2012; 13, Armenian, 2013; 14, Lipshultz, 2013; 15, Lubieniecka, 2013; 16, Visscher, 2013; 17, Vivenza, 2013; 18, Wang, 2014; 19, Wasielewski, 2014; 20, Aminkeng,2015; 21, Krajinovic, 2015; 22, Reichwagen, 2015; 23, Visscher, 2015; 24, Vulsteke, 2015; 25, Hertz, 2016; 26, Reinbolt, 2016; 27, Wang, 2016.

A, Rationale of Study; B, Selection and definition of outcome; C, Selection and comparability of comparison groups (0 if not applicable); D, Technical classification of the exposure; E, Non-technical classification of the exposure; F, Other source of bias; G, Sample size and power; H, A priopri planning of analyses; I, Statistical methods and control for confounding; J, Testing of assumption & inferences for genetic analyses; K, Appropriateness of inferences drawn from results. [1]G = Study of good quality, M =Study of moderate quality.

Supplementary Table 3: Genetic polymorphisms, association with ACT and HWE status

Gene	Nucleotide change	Amino acid change	SNP rsID	Study (Name, Year)	Association[2]	Deviation from HWE (exact test mid p-value)[1]
ABCA1 (NC_000009.11)	106738433G>A	None	rs3887137	Visscher,2015	**SNP is significantly associated with ACT in combined cohort OR = 2.28 (1.40 – 3.71), p = 9.5x10^{-4}**	No
ABCB1 (NC_000007.13)	g.87138532A>C	None	rs2235047	Visscher, 2013	No significant association between SNP and ACT OR = 1.79 (1.05 – 3.04), p = 0.036	No
	g.87138645A>G	Ile1145	rs1045642	Rossi, 2009	No significant association between genotypes and cardiac toxicity TT vs CT/CC OR = 1.16 (0.73 – 1.84), p = 0.515 CT/TT vs CC OR = 1.09 (0.69 – 1.73), p = 0.680	No
				Hertz, 2016	**CC vs CT/TT OR = 0.48 (0.23 – 1.00), p = 0.049**	No
	g.87179809C>T	Ser400Asn	rs2229109	Rossi, 2009	**AG vs GG OR = 1.89 (1.15 – 3.12), p = 0.010**	No
	g.87179601A>G	Gly412	rs1128503	Hertz, 2016	No significant association between genotype and cardiotoxicity GG vs GA/AA OR = 0.57 (0.27 – 1.21), p = 0.14	No
	g.87160618G>T	Ser893Thr	rs2032582		No significant association between genotype and cardiotoxicity GG vs GT/TT OR = 0.72 (0.36 – 1.43), p = 0.35	

Supplementary Table 3: Genetic polymorphisms, association with ACT and HWE status *(continued)*

Gene	Nucleotide change	Amino acid change	SNP rsID	Study (Name, Year)	Association[2]	Deviation from HWE (exact test mid p-value)[1]
ABCB4 (NC_000007.13)	g.87073775T>G	None	rs1149222	Visscher, 2012	**Risk variant**<br>**OR = 1.87 (1.20 – 2.92), p = 0.0054**	No
				Visscher, 2013	No significant association between SNP and ACT<br>OR = 1.36 (0.97 – 1.90), p = 0.075	No
				Hertz, 2016	No significant association between genotype and cardiotoxicity<br>TT vs GT/GG<br>OR = 1.06 (0.48 – 2.34), p = 0.14	No
				Sachidanandam, 2012	No significant association	NR
	g.87105795A>G	None	rs4148808	Visscher, 2013	**SNP is significantly associated with ACT in combined cohort**<br>**OR = 1.67 (1.15 – 2.43), p = 0.0073**	No
				Hertz, 2016	No significant association between genotype and cardiotoxicity<br>GG vs GA/AA<br>OR = 3.92 (0.34 – 45.39), p = 0.27	No
ABCB11 (NC_000002.12)	169479422A>G	None	rs10497346	Visscher, 2015	**SNP is significantly associated with ACT in combined cohort**<br>**OR = 2.23 (1.32 – 3.77), p = 0.0033**	No

Supplementary Table 3: Genetic polymorphisms, association with ACT and HWE status *(continued)*

Gene	Nucleotide change	Amino acid change	SNP rsID	Study (Name, Year)	Association[2]	Deviation from HWE (exact test mid p-value)[1]
ABCC1 (NC_000016.10)	g.15983430G>A	None	rs215060	Semsei, 2012	No significant reduction in LVFS after chemotherapy, p = 0.2	No
	g.15991778T>C	None	rs246219		No significant reduction in LVFS after chemotherapy, p = 0.6	No
	g.16108028G>A	None	rs11864374		No significant reduction in LVFS after chemotherapy, p = 0.2	No
	g.16120105G>A	None	rs6416666		No significant reduction in LVFS after chemotherapy, p = 0.3	No
	g.16141824C>T	None	rs3743527		**TT genotype is associated with lower mean LVFS, p = 0.001**	No
	g.16044465T>C	Val275=	rs246221	Semsei, 2012	**TT/TC genotypes are associated with lower mean LVFS, p = 0.027**	No
				Vulsteke, 2015	**SNP is associated with an asymptomatic LVEF decline > 10%, p = 0.038**	No
	g16079375G>T	Gly671Val	rs45511401	Semsei, 2012	No significant reduction in LVFS after chemotherapy, p = 0.3	No
				Reichwagen, 2015	No significant association between genotypes and cardiotoxicity GG vs GT/TT OR = 0.7 (0.2 – 2.5), p = 0.632	No
				Wojnowski, 2005	**GT/TT genotypes are associated with acute cardiotoxicity (p = 0.005) and chronic or acute cardiotoxicity (p = 0.029)**	No (0.14)
	g.6093318C>T	None	rs4148358	Semsei, 2012	No significant reduction in LVFS after chemotherapy, p = 0.3	No
	g.16076620G>T	None	rs4148350	Visscher, 2012	**Risk variant OR = 2.40 (1.33 – 4.33), p = 0.0040**	No
				Hertz, 2016	No significant association between genotype and cardiotoxicity GG vs GT/TT OR = 0.93 (0.21 – 4.04), p = 0.92	No

Supplementary Table 3: Genetic polymorphisms, association with ACT and HWE status *(continued)*

Gene	Nucleotide change	Amino acid change	SNP rsID	Study (Name, Year)	Association[2]	Deviation from HWE (exact test mid p-value)[1]
ABCC2 (NC_000010.10)	g.101611294G>A	Cys1515Tyr	rs8187710	Armenian, 2013	**At risk genotype GA/AA OR = 5.44 (1.92 – 13.84) FDR-adjusted p = 0.02**	No
				Wojnowski, 2005	No significant association between GA/AA genotypes and cardiotoxicity, p = 0.05	No
				Reichwagen, 2015	No significant association between genotypes and cardiotoxicity GG vs GA/AA OR = 1.3 (0.4 – 3.95), p = 0.669	No
	T>A	Val1188Glu	rs8187694	Visscher, 2012	No significant association p = 0.90	No
				Reichwagen, 2015	No significant association between genotypes and cardiotoxicity TT vs TA/AA OR = 1.1 (0.4 – 3.4), p = 0.822	No
				Wojnowski, 2005	**TA/AA genotypes are associated with chronic or acute cardiotoxicity, p = 0.05**	No
	g.101595996T>A	Val1085Glu	rs17222723	Rossi, 2009	No significant association between genotypes and cardiac toxicity AT/AA vs TT OR = 0.85 (0.50 – 1.43), p = 0.546	Yes
ABCC5 (NC_000003.11)	g.183737356A>T	None	rs7627754	Krajinovic, 2015	**Homozygote variant vs heterozygote + homozygote wt, EF reduction of 12%: p <0.0005 and SF reduction of 8%: p = 0.001 using t-test**	NR
ABCC6 (NC_000016.10)	g.16150272T>C	None	rs212097	Semsei, 2012	No significant reduction in LVFS after chemotherapy, p = 0.2	No

Supplementary Table 3: Genetic polymorphisms, association with ACT and HWE status *(continued)*

Gene	Nucleotide change	Amino acid change	SNP rsID	Study (Name, Year)	Association[2]	Deviation from HWE (exact test mid p-value)[1]
ABCC9 (NG_000012.11)	g.22017395G>C	Pro739Ala	rs201223488	Wasielweski, 2014	No causal relation between mutation and development of anthracycline-associated cardiomyopathy	No
	21908424G>C	None	rs11046217	Visscher, 2015	**SNP is significantly associated with ACT in combined cohort**<br>**OR = 2.67 (1.50 – 4.76), p = $9.9x10^{-4}$**	No
ABCC10 (NC_00006.11)	43468329A>G	None	rs1214763	Visscher, 2015	Yes **SNP is significantly associated with ACT in combined cohort**<br>**OR = 0.43 (0.24 – 0.79), p = 0.0035**	No
ABCG2 (NC_000004.11)	g.89052323G>T	Gln141Lyn	rs2231142	Rossi, 2009	No significant association between genotypes and cardiac toxicity<br>AC/AA vs CC<br>OR = 1.05 (0.70 – 1.57), p = 0.784	No
	g.89061114C>T	Val12Met	rs2231137		AG/AA vs GG<br>OR = 1.01 (0.56 – 1.80), p = 0.974	No
ACE (NC_000017.10)	Ins/Del	-	rs4340	Vivenza, 2013	Del/Del + Del/Ins vs Ins/Ins, p = 0.37 using Fischer's exact test	No
	g.61566031G>A	Thr202	rs4343	Armenian, 2013	At risk genotype AG/GG<br>OR = 1.28 (0.67 – 2.45)<br>FDR-adjusted p = 0.72	No
ADH7 (NC_000004.11)	g.100333267G>A	None	rs729147	Visscher, 2013	**SNP is significantly associated with ACT in combined cohort**<br>**OR = 1.43 (1.02 – 2.01), p = 0.041**	No
ADRB2 (NC_000005.10)	g.148206440G>A	Gly16Arg	rs1042713	Armenian, 2013	At risk genotype GG<br>OR = 0.60 (0.31 – 1.20)<br>FDR-adjusted p = 0.35	No

Supplementary Table 3: Genetic polymorphisms, association with ACT and HWE status *(continued)*

Gene	Nucleotide change	Amino acid change	SNP rsID	Study (Name, Year)	Association[2]	Deviation from HWE (exact test mid p-value)[1]
AGT (NC_000001.10)	g.230845977G>A	Thr174Met	rs4672	Vivenza, 2013	Homozygote variant vs heterozygote + homozygote wt, p = 0.70 using Pearson's chi-square test	No
	g.230845794A>G	Met235Thr	rs699	Vivenza, 2013	Homozygote variant + heterozygote vs homozygote wt, p = 0.31 using Fischer's exact test	No
				Armenian, 2013	At risk genotype GA/AA OR = 1.15 (0.57 – 2.30) FDR-adjusted p = 0.88	No
AGTR1 (NC_000003.11)	g.148459988A>C	None	rs5186	Vivenza, 2013	Homozygote variant + heterozygote vs homozygote wt, p = 0.31 using Pearson's chi-square test	No
				Armenian, 2013	At risk genotype CA/AA OR = 0.68 (0.37 – 1.25) FDR-adjusted p = 0.88	No
AKR1A1 (NC_000001.10)	g.46032311A>G	Asn52Ser	rs2229540	Lubieniecka, 2012	No significant association, p = 0.1667	No
AKR1C4 (NC_000010.10)	g.5260682C>G	Leu311Val	rs17134592	Lubieniecka, 2012	No significant association, p = 0.9556	No
	g.5244295G>A	None	rs7083869	Lubieniecka, 2013	**SNP is associated with LVEF drop p = 0.0045**	No
	g.5244441A>G	None	rs2151896		**SNP is associated with LVEF drop p = 0.045**	No
AKR7A2 (NC_000001.10)	g.19635011C>T	Ala142Thr	rs1043657	Lubieniecka, 2012	No significant association, p = 0.4379	No
CAT (NC_000011.10)	g.34438684C>T	None	rs1001179	Rajic, 2009	No significant correlation between SNP and late cardiac damage OR = 3.222 (0.341 – 30.426) p = 0.307	NR
	g.34439157C>T	None	rs10836235		**Heterozygote is correlated with late cardiac damage OR = 0.284 (0.093 – 0.867), p = 0.020**	NR

Supplementary Table 3: Genetic polymorphisms, association with ACT and HWE status *(continued)*

Gene	Nucleotide change	Amino acid change	SNP rsID	Study (Name, Year)	Association[2]	Deviation from HWE (exact test mid p-value)[1]
CBR1 (NC_000021.9)	g.37445313G>A	None	rs9024	Blanco, 2012	AA/GA vs GG<br>OR = (0.45 – 1.47), p = 0.49	No
				Hertz, 2016	No significant association between genotype and cardiotoxicity<br>GG vs GA/AA<br>OR = 0.45 (0.10 – 2.04), p = 0.30	No
				Reinbolt, 2016	No significant different between major allele (G) and minor allele (A), p = 0.480<br>No significant different between genotypes, p = 0.261	NR
				Sachidonandam, 2012	No significant association	NR
				Armenian, 2013	At risk genotype GG<br>OR = 1.51 (0.76 – 3.03)<br>FDR-adjusted p = 0.46	No

Supplementary Table 3: Genetic polymorphisms, association with ACT and HWE status *(continued)*

Gene	Nucleotide change	Amino acid change	SNP rsID	Study (Name, Year)	Association[2]	Deviation from HWE (exact test mid p-value)[1]
CBR3 (NC_000021.8)	g.37518706G>A	Val244Met	rs1056892	Blanco, 2008	GG vs AA<br>OR = 5.63 (0.80 – 39.57), p = 0.083<br>GA vs AA<br>OR = 3.66 (0.64 – 21.07), p = 0.15	No
				Lubieniecka, 2012	No significant association, p = 0.6988	No
				Armenian, 2013	At risk genotype GG<br>OR = 1.08 (0.59 – 1.87)<br>FDR-adjusted p = 0.88	No
				Visscher, 2015	No significant association between SNP and ACT<br>OR = 0.93, p = 0.67	No
				Blanco, 2012	**AA/GA vs GG**<br>**OR = 1.79 (1.08 – 2.96), p = 0.02**	No
				Volkan-Salanci, 2012	Presence of A allele is associated with deterioration in cardiac function.	No
				Hertz, 2016	**AA vs GA/GG**<br>**OR = 6.19 (0.08 – 19.76), p = 0.002**<br>**GG vs GA/AA**<br>**OR = 2.50 (1.22 – 5.11), p = 0.012**	No
				Reinbolt, 2016	No significant different between major allele (G) and minor allele (A), p = 0.395<br>No significant different between genotypes, p = 0.556	NR
	g.37507501G>A	Cys4Tyr	rs8133052	Lubieniecka, 2012	No significant association, p = 0.7788	No
	g.37512565A>G	None	rs10483032	Lubieniecka, 2013	**SNP is associated with LVEF drop**<br>**p = 0.0045**	No

Supplementary Table 3: Genetic polymorphisms, association with ACT and HWE status *(continued)*

Gene	Nucleotide change	Amino acid change	SNP rsID	Study (Name, Year)	Association[2]	Deviation from HWE (exact test mid p-value)[1]
CELF4 (NC_000018.10)	g.37497065G>A	None	rs1786814	Wang, 2016	**GG genotype is associated with anthracycline-related cardiomyopathy OR = 10.16 (3.8 – 27.3), p < 0.001 at cumulative anthracycline exposure of > 300mg/m^2**	No
COL1A2 (NC_000007.13)	g.93881175C>G	Pro549Ala	rs42524	Visscher, 2015	**SNP is significantly associated with ACT in combined cohort OR = 1.79 (1.24 – 2.57), p = 0.0020**	No
CYBA (NC_000016.10)	242C>T	His72Tyr	rs4673	Visscher, 2012	No significant association p = 0.63	No
				Reichwagen, 2015	No significant association between genotypes and cardiotoxicity CC vs CT/TT OR = 1.8 (0.9 – 3.6), p = 0.090	No
				Hertz, 2016	No significant association between genotype and cardiotoxicity GG vs GA/AA OR = 1.31 (0.66 – 2.59), p = 0.45	No
				Wojnowski, 2005	**CT/TT genotypes are associated with chronic or acute cardiotoxicity, p = 0.01**	No
				Rossi, 2009	**TT vs CT/CC OR = 1.86 (1.15 – 2.99), p = 0.010**	No
				Armenian, 2013	At risk genotype GA/AA OR = 1.29 (0.72 – 2.44) FDR-adjusted p = 0.65	No
CYP1A2 (NC_000015.10)	g.74746892T>G	None	rs2069522	Lubieniecka, 2013	**SNP is associated with LVEF drop p = 0.004**	No
	g.74749000T>G	None	rs2069526		**SNP is associated with LVEF drop p = 0.0045**	No
	g.74753351T>C	None	rs4646427		**SNP is associated with LVEF drop p = 0.0045**	No

Supplementary Table 3: Genetic polymorphisms, association with ACT and HWE status *(continued)*

Gene	Nucleotide change	Amino acid change	SNP rsID	Study (Name, Year)	Association[2]	Deviation from HWE (exact test mid p-value)[1]
CYP2B6 (NC_000019.10)	g.41023115G>A	None	rs7255904	Lubieniecka, 2013	**SNP is associated with LVEF drop p = 0.0475**	No
CYP2F1 (NC_000019.10)	g.41113670T>C	None	rs1709115	Lubieniecka, 2013	**SNP is associated with LVEF drop p = 0.042**	No
CYP2J2 (NC_000001.10)	60087084A>C	None	rs2294950	Visscher, 2015	**SNP is significantly associated with ACT in combined cohort OR = 0.39 (0.21 – 0.74), p = 0.0014**	No
CYP3A4 (NC_000007.13)	g.99366316G>A	None	rs35599367	Hertz, 2016	No significant association between genotype and cardiotoxicity GG vs GA/AA OR = 0.65 (0.09 – 4.73), p = 0.67	No
CYP3A5 (NC_000007.13)	g.99270539C>T	None	rs776746	Hertz, 2016	No significant association between genotype and cardiotoxicity GG vs GA/AA OR = 2.17 (0.80 – 5.94), p = 0.13	No
CYP4B1 (NC_000001.10)	g.47265776A>G	None	rs837400	Lubieniecka, 2013	**SNP is associated with LVEF drop p = 0.021**	No
	g.47283505G>T	None	rs4646495		**SNP is associated with LVEF drop p = 0.041**	No
CYP4F11 (NC_000019.10)	g.15906807G>A	None	rs8112732	Lubieniecka, 2013	**SNP is associated with LVEF drop p = 0.0235**	No
	g.15912567A>G	None	rs12610962		**SNP is associated with LVEF drop p = 0.028**	No
	g.15913964A>G	None	rs2072270		**SNP is associated with LVEF drop p = 0.0285**	No
	g.15921833A>C	None	rs11086012		No significant associated between SNP and LVEF drop, p = 0.051	No
	g.15906196G>A	None	rs2108623	Visscher, 2013	No significant association between SNP and ACT OR = 0.77 (0.57 – 1.04), p = 0.084	No
CYP11B2 (NC_000008.10)	g.143999600A>G	None	rs1799998	Vivenza, 2013	Homozygote variant + heterozygote vs homozygote wt, p = 0.31 using Fischer's exact test	No

Supplementary Table 3: Genetic polymorphisms, association with ACT and HWE status *(continued)*

Gene	Nucleotide change	Amino acid change	SNP rsID	Study (Name, Year)	Association[2]	Deviation from HWE (exact test mid p-value)[1]
FMO2 (NC_000001.10)	37236730T>A	Asp36Gly	rs2020870	Visscher, 2012	**Protective variant OR = 0.14 (0.03 – 0.59), p = 4.2x10^{-4}**	No
FMO3 (NC_000001.10)	g.171080080G>A	Val257Met	rs1736557	Visscher, 2013	**SNP is significantly associated with ACT in combined cohort OR = 0.47 9 0.25 – 0.87), p = 0.011**	No
GPX3 (NC_000005.10)	150395291G>C	None	rs2233302	Visscher, 2015	**SNP is significantly associated with ACT in combined cohort OR = 0.40 (0.22 – 0.73), p = 0.0011**	No
GSTA1 (NC_000006.11)	NR	NR	NR	Weiss, 2006	No significant associations between genotype and cardiac events	No (0.34)
GSTA2 (NC_000006.11)	52725690C>G	Ser112Thr	rs2180314	Visscher, 2015	**SNP is significantly associated with ACT in combined cohort OR = 0.62 (0.45 – 0.86), p = 0.0036**	No
GSTM1 (NC_000001.10)	NA	NA	NA	Weiss, 2006	No significant associations between genotype and cardiac events	NR
	NA	NA	NA	Rajic, 2009	No significant correlation between SNP and late cardiac damage OR = 1.089 (0.312 – 3.798) p = 0.895	NR
	NA	NA	NA	Rossi, 2009	No significant association between genotypes and cardiac toxicity Null vs wt OR = 0.92 (0.62 – 1.36), p = 0.681	No
	NA	NA	NA	Vivenza, 2013	Present (+/- and +/+) vs Null (-/-), p = 0.147 using Fisher's exact test	Yes
GSTT1 (NC_000022.10)	NA	NA	NA	Weiss, 2006	No significant associations between genotype and cardiac events	NR
	NA	NA	NA	Rajic, 2009	No significant correlation between SNP and late cardiac damage OR = 6.222 (0.726 – 53.348) p = 0.063	NR
	NA	NA	NA	Vivenza, 2013	Present (+/- and +/+) vs Null (-/-), p = 0.687 using Fisher's exact test	Yes

Supplementary Table 3: Genetic polymorphisms, association with ACT and HWE status *(continued)*

Gene	Nucleotide change	Amino acid change	SNP rsID	Study (Name, Year)	Association[2]	Deviation from HWE (exact test mid p-value)[1]
GSTM3 (NC_000001.11)	110075064 A>G	None	rs12059276	Visscher, 2015	**SNP is significantly associated with ACT in combined cohort OR = 0.36 (0.17 – 0.76), p = 0.0031**	No
GSTP1 (NC_000011.10)	g.67585218A>G	Ile105Val	rs1695	Volkan-Salanci, 2012	Presence of G allele is associated with deterioration in cardiac function.	No
				Rossi, 2009	**GG vs AG/AA OR = 1.83 (1.12 – 3.01), p = 0.015**	No
				Windsor, 2012	**Variants are associated with increased risk of cardiotoxicity OR = 4.8 (1.4 – 16.4), p = 0.011**	No
				Vivenza, 2013	Homozygote variant + heterozygote vs homozygote wt, p = 0.20 using Pearson's chi-square test	Yes
	g.67586108C>T	Ala114Val	rs1138272	Rossi, 2009	No significant association between genotypes and cardiac toxicity CT vs CC OR = 0.99 (0.52 – 1.87), p = 0.978	No
HAS3 (NC_000016.10)	g.69109674A>C	Ala93=	rs2232228	Wang, 2014	**Presence of allele A increase the risk of cardiomyopathy with the increase of cumulative anthracycline exposure.**	No

Supplementary Table 3: Genetic polymorphisms, association with ACT and HWE status *(continued)*

Gene	Nucleotide change	Amino acid change	SNP rsID	Study (Name, Year)	Association[2]	Deviation from HWE (exact test mid p-value)[1]
HFE (NC_000006.11)	g.26093141G>A	Cys282Tyr	rs1800562	Armenian, 2013	At risk genotype GA/AA<br>OR = 0.30 (0.05 – 1.23)<br>FDR-adjusted p = 0.28	No
				Lipshultz, 2013	**Heterozygote is associated with multiple elevations in cTnT concentration**<br>**OR = 7.23 (1.78 – 29.4), p = 0.006**	NR
	g.26091179C>G	His63Asp	rs1799945	Armenian, 2013	**At risk genotype CG/GG**<br>**OR = 2.58 (1.27 – 5.20)**<br>**FDR-adjusted p = 0.03**	No
				Lipshultz, 2013	No association between heterozygote or homozygote with cardiac markers	NR
HNMT (NC_000002.11)	g.13876039C>A	None	rs17583889	Visscher, 2012	**Risk variant**<br>**OR = 1.91 (1.21 – 3.02), p = 0.0057**	No
				Visscher, 2013	**SNP is significantly associated with ACT in combined cohort**<br>**OR = 1.67 (1.15 – 2.41), p = 0.0073**	No
				Sachidanandam, 2012	Presence of homozygote variant (high risk) and heterozygote (intermediate risk)	NR
	g.138780932T>C	None	rs17645700	Visscher, 2013	**SNP is significantly associated with ACT in combined cohort**<br>**OR = 0.56 (0.37 – 0.86), p = 0.0054**	No
HSD17B2 (NC_000016.10)	g.82028304T>G	None	rs16956248	Lubieniecka, 2013	**SNP is associated with LVEF drop**<br>**p = 0.023**	No
	g.820829177T>A	None	rs13333826		**SNP is associated with LVEF drop**<br>**p = 0.024**	No
	g.82054058C>T	None	rs7196087		**SNP is associated with LVEF drop**<br>**p = 0.028**	No
	g.82079586C>T	None	rs2955159		**SNP is associated with LVEF drop**<br>**p = 0.028**	No
	g.82072694G>A	None	rs2966245		**SNP is associated with LVEF drop**<br>**p = 0.0305**	No

Supplementary Table 3: Genetic polymorphisms, association with ACT and HWE status *(continued)*

Gene	Nucleotide change	Amino acid change	SNP rsID	Study (Name, Year)	Association[2]	Deviation from HWE (exact test mid p-value)[1]
HSD17B4 (NC_000005.9)	g.119502865G>T	None	rs257970	Lubieniecka, 2013	**SNP is associated with LVEF drop p = 0.003**	No
	g.119502373G>A	None	rs2636968		**SNP is associated with LVEF drop p = 0.007**	No
NCF4 (NC_000022.10)	g.37256846GA>G	None	rs1883112	Visscher, 2012	No significant association, p = 0.76	No
				Hertz, 2016	No significant association between genotype and cardiotoxicity AA vs GA/GG OR = 1.44 (0.51 – 4.07), p = 0.49	No
				Reichwagen, 2015	No significant association between genotypes and cardiotoxicity GG/GA vs AA OR = 0.6 (0.3 – 1.4), p = 0.280	No
				Armenian, 2013	At risk genotype AA OR = 0.06 (0.54 – 2.13) FDR-adjusted p = 0.88	No
				Rossi, 2009	**AG/GG vs AA OR = 0.39 (0.24 – 0.64), p = $1.4x10^{-4}$**	No
				Wojnowski, 2005	**AA genotype is associated with chronic cardiotoxicity (p = 0.013) and chronic or acute cardiotoxicity (p = 0.031)**	No
NOS3 (NC_000007.13)	g.150696111T>G	p.Asp298Glu	rs1799983	Krajinovic, 2015	Protective effect of the TT genotype on ejection fraction (p = 0.02	NR
NQO1 (NC_000016.9)	g.69745145C>T	Pro187Ser	rs1800566	Armenian, 2013	At risk genotype CT/CC OR = 0.88 (0.23 – 3.42) FDR-adjusted p = 0.88	No
				Blanco, 2008	CC vs TT OR = 1.26 (0.14 – 11.39), p = 0.84 CT vs TT OR = 0.65 (0.06 – 6.77), p = 0.72	No

Supplementary Table 3: Genetic polymorphisms, association with ACT and HWE status *(continued)*

Gene	Nucleotide change	Amino acid change	SNP rsID	Study (Name, Year)	Association[2]	Deviation from HWE (exact test mid p-value)[1]
NR1I2 (NC_000003.11)	g.119501039C>A	None	rs1523127	Hertz, 2016	No significant association between genotype and cardiotoxicity TT vs TG/GG OR = 0.76 (0.37 – 1.54), p = 0.44	No
	g.119530858G>A	None	rs3732357		No significant association between genotype and cardiotoxicity AA vs GA/GG OR = 1.44 (0.76 – 2.70), p = 0.26	No
	g.119499507T>C	None	rs1523130		No significant association between genotype and cardiotoxicity GG vs GA/AA OR = 1.11 (0.57 – 2.17), p = 0.76	No
POR (NC_000007.13)	g.75589903A>G	None	rs2868177	Lubieniecka, 2013	**SNP is associated with LVEF drop p = 0.0025**	No
	g.75606109G>A	None	rs13240755		**SNP is associated with LVEF drop p = 0.0035**	No
	g.75607608C>T	None	rs4732513		**SNP is associated with LVEF drop p = 0.004**	No
	g.75601169G>A	None	rs6953065		SNP is associated with LVEF drop p = 0.0445	No

Supplementary Table 3: Genetic polymorphisms, association with ACT and HWE status *(continued)*

Gene	Nucleotide change	Amino acid change	SNP rsID	Study (Name, Year)	Association[2]	Deviation from HWE (exact test mid p-value)[1]
RAC2 (NC_000022.11)	g.37236730T>A	None	rs13058338	Armenian, 2013	**At risk genotype TA/AA OR = 2.61 (1.46 – 4.69) FDR-adjusted p = 0.02**	No
				Wojnowski, 2005	**TA/AA genotypes are associated with acute cardiotoxicity (p = 0.005) and chronic or acute cardiotoxicity (p = 0.04)**	No (0.95)
				Rossi, 2009	**AA vs AT/TT OR = 1.84 (1.10 – 3.10), p = 0.019**	No
				Reichwagen, 2015	No significant association between genotypes and cardiotoxicity GG vs GT/TT OR = 0.7 (0.2 – 2.5), p = 0.632	No
				Hertz, 2016	No significant association between genotype and cardiotoxicity TT vs TA/AA OR = 1.8 (0.9 – 3.7), p = 0.077	No
				Visscher, 2012	No significant association p = 0.13	
RARG (NC_000012.11)	g.53605545G>A	Ser4271Leu	rs2229774	Aminkeng, 2015	All population OR 4.7 (2.7-8.3), p = 4.3 x 10^{-11}	No
SERPINA6 (NC_000014.8)	93848406G>A	None	rs10144771	Visscher, 2015	**SNP is significantly associated with ACT in combined cohort OR = 1.72 (1.19 – 2.50), p = 0.0042**	No
SLC10A2 (NC_000013.10)	g.103714254G>A	None	rs9514091	Visscher, 2012	**Protective variant OR = 0.43 (0.23 – 0.78), p = 0.0033**	No
				Visscher, 2013	**SNP is significantly associated with ACT in combined cohort OR = 0.57 (0.38 – 0.87)**	No
	g.103723722G>A	None	rs7319981	Visscher, 2013	**SNP is significantly associated with ACT in combined cohort OR = 0.66 (0.47 – 0.93), p = 0.016**	No

Supplementary Table 3: Genetic polymorphisms, association with ACT and HWE status *(continued)*

Gene	Nucleotide change	Amino acid change	SNP rsID	Study (Name, Year)	Association[2]	Deviation from HWE (exact test mid p-value)[1]
SLC13A3 (NC_000020.10)	44693250A>G	None	rs2425886	Visscher, 2015	**SNP is significantly associated with ACT in combined cohort OR = 1.75 (1.20 – 2.57), p = 0.0037**	No
SLC15A1 (NC_000013.10)	98153602G>C	None	rs8001466	Visscher, 2015	**SNP is significantly associated with ACT in combined cohort OR = 2.02 (1.25 – 3.26), p = 0.0042**	No
SLC22A2 (NC_000006.11)	g.160670282A>C	Ser270Ala	rs316019	Visscher, 2013	No significant association between SNP and ACT OR = 0.75 9 0.46 – 1.21), p = 0.23	No
SLC22A7 (NC_000006.12)	43389166A>G	None	rs4149178	Visscher, 2015	**SNP is significantly associated with ACT in combined cohort OR = 0.45 (0.26 – 0.75) p = 0.0013**	No
SLC22A16 (NC_000006.11)	g.110778128T>C	His49Arg	rs714368	Hertz, 2016	No significant association between genotype and cardiotoxicity AA vs GA/GG OR = 0.78 (0.31 – 1.96), p = 0.60	No
	g.110777962A>G	Asn104	rs6907567		No significant association between genotype and cardiotoxicity AA vs GA/GG OR = 0.74 (0.30 – 1.83), p = 0.51	No
	g.110763875A>G	Val252Ala	rs723685		No significant association between genotype and cardiotoxicity TT vs TC/CC OR = 0.44 (0.10 – 2.03), p = 0.29	No
	g.110760008A>G	Met377Thr	rs12210538		No significant association between genotype and cardiotoxicity AA vs GA/GG OR = 1.30 (0.63 – 2.71), p = 0.48	No

Supplementary Table 3: Genetic polymorphisms, association with ACT and HWE status *(continued)*

Gene	Nucleotide change	Amino acid change	SNP rsID	Study (Name, Year)	Association[2]	Deviation from HWE (exact test mid p-value)[1]
SLC22A17 (NC_000014.8)	22884409G>A	None	rs4982753	Visscher, 2015	**SNP is significantly associated with ACT in combined cohort OR = 0.50 (0.33 – 0.75), p = 4.4x10^{-4}**	No
	23814995A>T	None	rs11625724		**SNP is significantly associated with ACT in combined cohort OR = 1.63 (0.0020)**	No
	23812237G>C	None	rs12882406		**SNP is significantly associated with ACT after adjusting the effect of rs4982753 OR = 1.52, p = 0.042**	No
	23816998T>C	None	rs12896494		**SNP is significantly associated with ACT after adjusting the effect of rs4982753 OR = 0.65, p = 0.031**	No
SLC28A1 (NC_000015.10)	g.84909044T>C	None	rs2305364	Visscher, 2013	**SNP is significantly associated with ACT in combined cohort OR = 1.60 (1.18 – 2.17), p = 0.0020**	No
	g.84904404A>C	None	rs2290271	Visscher, 2013	**SNP is significantly associated with ACT in combined cohort OR = 0.66 (0.48 – 0.91)**	No
SLC28A3 (NC_000009.11)	g.86900926G>A	Leu461=	rs7853758	Visscher, 2012	**Protective variant OR = 0.31 (0.16 – 0.60), p = 1.0x10^{-4}**	No
				Visscher, 2013	**SNP is significantly associated with ACT in combined cohort OR = 0.36 (0.22 – 0.60), p = 1.6 x 10^{-5}**	No
				Reichwagen, 2015	No significant association between genotypes and cardiotoxicity GG vs GA/AA OR = 1.4 (0.7 – 3.0), p = 0.393	No
				Hertz, 2016	No significant association between genotype and cardiotoxicity GG vs GA/AA OR = 0.55 (0.16 – 1.91), p = 0.35	No

Supplementary Table 3: Genetic polymorphisms, association with ACT and HWE status *(continued)*

Gene	Nucleotide change	Amino acid change	SNP rsID	Study (Name, Year)	Association[2]	Deviation from HWE (exact test mid p-value)[1]
SLC28A3 (NC_000009.11)	g.86946417A>C	None	rs4877847	Visscher, 2012	**Protective variant OR = 0.60 (0.41 – 0.89), p = 0.0092**	No
				Visscher, 2013	**SNP is significantly associated with ACT in combined cohort OR = 0.73 (0.54 – 0.98), p = 0.037**	
	g.86909550G>A	None	rs885004	Visscher, 2013	**SNP is significantly associated with ACT in combined cohort OR = 0.34 (0.20 – 0.60), p = 3.0×10^{-5}**	No
SLCO4C1 (NC_000005.10)	g.101633540G>A	None	rs2600834	Visscher, 2015	**SNP is significantly associated with ACT in combined cohort OR = 1.80 (1.26 – 2.57), p = 0.0011**	No
SLCO6A1 (NC_000005.10)	g.101779552A>G	None	rs12658397	Visscher, 2015	**SNP is significantly associated with ACT in combined cohort OR = 1.68 (1.20 – 2.35), p = 0.0025**	No
SOD2 (NC_000006.11)	g.159981080G>A	None	rs7754103	Visscher, 2015	**SNP is significantly associated with ACT in combined cohort OR = 0.32 (0.15 – 0.72), p = 0.0024**	No
	g.160113872A>G	Val16Ala	rs4880	Rajic, 2009	No significant correlation between SNP and late cardiac damage OR = 0.917 (0.328 – 2.562) p = 0.907	NR
				Armenian, 2013	At risk genotype GA/AA OR = 1.79 (0.90 – 3.38) FDR-adjusted p = 0.28	No
SPG7 (NC_000016.10)	g.89549357T>G	None	rs2019604	Visscher, 2012	**Protective variant OR = 0.39 (0.20 – 0.76), p = 0.0021**	No
				Visscher, 2013	**SNP is significantly associated with ACT in combined cohort OR = 0.56 (0.35 – 0.90), p = 0.012**	No

Supplementary Table 3: Genetic polymorphisms, association with ACT and HWE status *(continued)*

Gene	Nucleotide change	Amino acid change	SNP rsID	Study (Name, Year)	Association[2]	Deviation from HWE (exact test mid p-value)[1]
SULT2B1 (NC_000019.10)	g.48588977C>A	None	rs10426377	Visscher, 2013	**SNP is significantly associated with ACT in combined cohort OR =0.56 (0.38 – 0.81), p = 0.0015**	No
	48589173A>G		rs10426628	Visscher, 2015	**SNP is significantly associated with ACT in combined cohort OR = 1.92 (1.34 – 2.73), p = $3.2x10^{-4}$**	No
TOP2B (NC_000003.11	g.24929802C>A	None	rs10865801	Hertz, 2016	No significant association between genotype and cardiotoxicity CC vs CT/TT OR = 1.32 (0.67 – 2.61), p = 0.43	No
TP53 (NC_000017.10)	g.757972G>C	Pro72Arg	rs1042522	Rossi, 2009	No significant association between genotypes and cardiac toxicity CG/CC vs GG OR = 1.20 (0.81 – 1.78), p = 0.357	No
				Vivenza, 2013	Homozygote variant + heterozygote vs homozygote wt, p = 0.33 using Pearson's chi-square test	Yes
UGT1A6 (NC_000002.11)	g.234601669T>G	Ser7Ala	rs6759892	Visscher, 2012	**Risk variant OR = 1.77 (1.20 – 2.61), p = 0.0038**	No
				Visscher, 2013	**SNP is significantly associated with ACT in combined cohort OR = 1.43 (1.05 – 1.94), p = 0.022**	No
	g.234602277G>T	Val209=	rs17863783	Visscher, 2013	**SNP is significantly associated with ACT in combined cohort OR = 4.30 (1.97 – 9.36), p = $2.4x10^{-4}$**	No
				Hertz, 2016	Only homozygote wt were presence	No
	g.234593117G>T	None	rs4261716	Visscher, 2013	**SNP is significantly associated with ACT in combined cohort OR = 1.44 (1.06 – 1.95), p = 0.018**	
DSG2 (NC_000018.10)	g.31521193T>G	Val158Gly	rs191143292	Wasielewski, 2014	No causal relation between mutation and cardiomyopathy	No

Supplementary Table 3: Genetic polymorphisms, association with ACT and HWE status *(continued)*

Gene	Nucleotide change	Amino acid change	SNP rsID	Study (Name, Year)	Association[2]	Deviation from HWE (exact test mid p-value)[1]
JUP (NC_000017.10)	g.39913771C>T	Val648Ile	rs143043662	Wasielewski, 2014	No causal relation between mutation and cardiomyopathy	No
VCL (NC_000010.10)	g.75873961C>T	Ala990Val	rs150595117	Wasielewski, 2014	No causal relation between mutation and cardiomyopathy	No
MYH7 (NC_000014.8)	NR	Asp(545, 955)Asn	NR	Wasielewski, 2014	No causal relation between mutation and cardiomyopathy	No
	NR	Tyr1375	NR		No causal relation between mutation and cardiomyopathy	No
TTN (NC_000002.11)	NR	Ser31346Leu	NR	Wasielewski, 2014	No causal relation between mutation and cardiomyopathy	No
	g.179602871T>C	Tyr4453Cys	rs371552518		No causal relation between mutation and cardiomyopathy	No
	NR	Glu10855dup	NR		No causal relation between mutation and cardiomyopathy	No
DSP (NC_000006.11)	NR	Arg1425Lys	NR	Wasielewski, 2014	No causal relation between mutation and cardiomyopathy	No
PKP2 (NC_000012.11)	g.32994058A>C	Ile531Ser	rs147240502	Wasielewski, 2014	No causal relation between mutation and cardiomyopathy	No
XDH (NC_000002.11)	g.31460174G>A	Val279	rs4407290	Visscher, 2015	**SNP is significantly associated with ACT in combined cohort OR = 0.18 (0.04 – 0.79), p = 0.0039**	No
	g.31460632A>G	None	rs2236168		**SNP is significantly associated with ACT in combined cohort OR = 1.68 (1.21 – 2.34), p = 0.0017**	No

FDR, false discovery rate; HWE, Hardy-Weinberg Equilibrium; NR, not reported
[1]In studies that had not reported for HWE testing we used the BGI Cognitive Genomics (available at https://www.cog-genomics.org/software/stats to test for the deviation. A mid p-value of less than 0.05 is considered statistical deviation from HWE.
[2]Significant associations are in bold (p<0.05)
